# Development and comparative pharmacokinetic evaluation of iodine-125- and fluorine-18-labelled analogues of 4-(benzo[*d*]thiazol-2-yl)phenylboronic acid for boron neutron capture therapy

**DOI:** 10.1039/d6ra07719f

**Published:** 2026-11-02

**Authors:** Changkeun Im, Mah Rukh Zia, Iqra Bibi, Kyung Jun Kang, Jung Young Kim, Ho Young Kim, Ji-Ae Park

**Affiliations:** a Division of Applied RI, Korea Institute of Radiological & Medical Sciences (KIRAMS) 75 Nowon-ro, Nowon-gu Seoul 01812 Republic of Korea areas2001@kirams.re.kr jpark@kirams.re.kr; b Radiological and Medico-Oncological Sciences, University of Science and Technology (UST) 217, Gajeong-ro, Yuseong-gu Daejeon 34113 Republic of Korea

## Abstract

Boron neutron capture therapy (BNCT) requires accurate assessment of boron biodistribution to support treatment planning. Radiolabelled analogues of 4-(benzo[*d*]thiazol-2-yl)phenylboronic acid (BTPB) were developed to investigate how radiohalogen substitution influences *in vivo* distribution and to explore their potential for imaging BTPB-related distribution characteristics. The aryl boronic acid moiety of BTPB was replaced with iodine-125 through copper-mediated iododeboronation to generate [^125^I]IPBT, or with fluorine-18 through copper-mediated nucleophilic radiofluorination to produce [^18^F]FPBT. Iododeboronation afforded [^125^I]IPBT with an isolated radiochemical yield of 28.39 ± 5.12%, whereas radiofluorination afforded [^18^F]FPBT with a lower decay-corrected isolated yield of 3.10 ± 1.35%. Despite comparable radiochemical purity, lipophilicity, and *in vitro* radiolabel stability, the two analogues showed distinct *in vivo* distribution profiles. Biodistribution analysis showed that [^18^F]FPBT exhibited selected temporal distribution characteristics relevant to BTPB imaging, including early brain uptake and a time-dependent increase in tumour-to-brain contrast, whereas [^125^I]IPBT showed a distinct organ distribution pattern consistent with possible *in vivo* deiodination. In an orthotopic glioblastoma model, [^18^F]FPBT and BTPB exhibited a qualitatively similar directional change toward increased tumour-to-brain contrast within their respective observation periods. However, because the two compounds were evaluated using different administered masses, analytical methods, and sampling intervals, these findings do not establish pharmacokinetic equivalence. These results demonstrate that radiohalogen substitution substantially influences the *in vivo* behaviour of BTPB-derived analogues and support further investigation of [^18^F]FPBT as a candidate positron emission tomography imaging analogue for assessing intracranial distribution characteristics relevant to BTPB. Validation under matched experimental conditions is required to determine whether [^18^F]FPBT can quantitatively reflect BTPB-derived boron distribution.

## Abstract 2

Radiohalogenated BTPB analogues exhibit distinct *in vivo* distributions. [^18^F]FPBT shows early brain uptake and increasing tumour-to-brain contrast, supporting further evaluation as a candidate PET imaging analogue.

## Graphical abstract

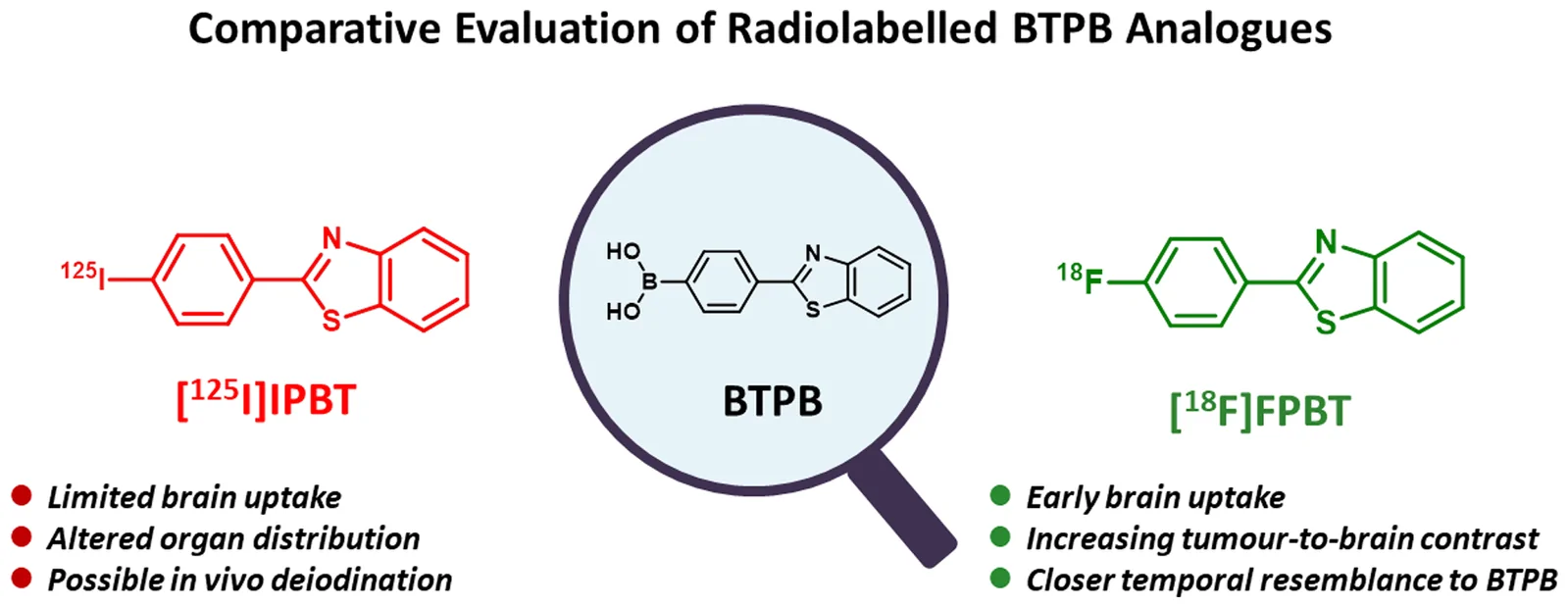

## 1. Introduction

Boron neutron capture therapy (BNCT) is a binary radiotherapeutic modality that relies on the nuclear interaction between boron-10 (^10^B) and low-energy thermal neutrons to achieve spatially confined cytotoxicity.^1,2^ Upon thermal-neutron capture, ^10^B undergoes the ^10^B(*n*,*α*)^7^Li reaction, generating high linear energy transfer (LET) α-particles and recoiling lithium nuclei.^2,3^ These densely ionising particles have path lengths comparable to the diameter of a single cell, approximately 5–10 µm, resulting in highly localised energy deposition and an elevated relative biological effectiveness (RBE).^3–7^ In principle, this microdosimetric selectivity enables preferential killing of malignant cells, provided that sufficient boron accumulation is achieved in tumour tissue relative to the surrounding normal tissues.^4^

Accordingly, the therapeutic index of BNCT is determined not only by neutron-flux characteristics but also by the pharmacokinetics and spatial distribution of the administered boron carrier.^5,6^ Quantitative parameters, including intratumoural distribution heterogeneity and temporal clearance kinetics, directly influence absorbed-dose estimation and the accuracy of treatment planning.^5,6^ Non-invasive imaging modalities capable of reliably reflecting *in vivo* boron biodistribution are therefore important for the clinical implementation of BNCT.^6^

Among the boron-delivery agents investigated clinically, 4-borono-l-phenylalanine (BPA) remains the most widely used.^8,9^ BPA, a boronated analogue of phenylalanine, is transported into tumour cells predominantly through l-type amino acid transporter 1 (LAT1), which is frequently overexpressed in malignant tissues.^10,11^ Radiolabelled analogues of BPA have consequently been developed using several radionuclide platforms to facilitate imaging-based evaluation of its biodistribution.^12–15^ Carbon-11-labelled BPA ([^11^C]BPA) retains the chemical structure of the parent compound and is therefore an ideal tracer in terms of structural fidelity; however, the short physical half-life of carbon-11, approximately 20 min, limits logistical flexibility and widespread clinical use.^14^ Iodine-123-labelled derivatives have also been investigated for single-photon emission computed tomography (SPECT),^14^ although modifications of the aromatic framework and limitations in quantitative reproducibility have restricted their broader adoption.^9^

In current clinical practice, positron emission tomography (PET) using 4-borono-2-[^18^F]fluoro-l-phenylalanine ([^18^F]FBPA) has emerged as the principal non-invasive imaging surrogate for estimating BPA biodistribution.^10–12^ Although [^18^F]FBPA is not structurally identical to BPA,^9^ comparative pharmacokinetic studies have demonstrated sufficient concordance in tumour uptake and tissue distribution to support its application in BNCT treatment planning.^11,12^ This experience illustrates an important principle in BNCT imaging: structural similarity and radiochemical accessibility alone do not necessarily ensure biological fidelity. Radiolabelled analogues intended as imaging surrogates must therefore undergo careful biological and pharmacokinetic validation to establish the extent to which their *in vivo* distribution reflects that of the parent boron agent.

In a previous study, we developed 4-(benzo[*d*]thiazol-2-yl)phenylboronic acid (BTPB) as a benzothiazole-based boron agent for BNCT.^16^ In contrast to BPA, BTPB exhibited favourable intracranial distribution characteristics, including early blood–brain barrier (BBB) penetration and prolonged boron retention in brain tissue.^16^ Its accumulation in tumour tissue further suggested that BTPB may be a promising alternative boron carrier for future BNCT applications.^16^ The intracranial distribution observed in that study is consistent with passive diffusion across the BBB, which would be advantageous for delivering boron to intracranial tumours.

From a chemical perspective, BTPB contains an aryl boronic acid moiety that is amenable to transition-metal-mediated halodeboronation, enabling the preparation of radioiodinated and radiofluorinated analogues.^17–22^ However, the choice of radiohalogen and labelling chemistry may influence not only radiosynthetic efficiency but also *in vivo* metabolic stability, susceptibility to dehalogenation, and organ-specific clearance.^23,24^ Consequently, even structurally related radiolabelled analogues cannot be assumed to exhibit pharmacokinetic behaviour equivalent to that of the parent compound. Systematic evaluation of radiolabelled phenylbenzothiazole derivatives is therefore required to determine which *in vivo* distribution characteristics of BTPB are retained after radiohalogen substitution and whether such analogues warrant further development as candidate imaging probes.

BTPB and its radiohalogenated analogues IPBT and FPBT share a common 2-phenylbenzothiazole scaffold, despite differences in the *para*-substituent on the phenyl ring. This structural similarity raises the possibility that the three compounds retain common molecular recognition characteristics. Previous studies of benzothiazole derivatives have reported aryl hydrocarbon receptor (AhR)-dependent biological activity, suggesting that AhR may be a relevant molecular interaction partner for this scaffold.^25,26^ Accordingly, molecular docking was included as an exploratory analysis to compare the predicted binding modes of BTPB, IPBT, and FPBT within the AhR ligand-binding domain and to assess whether the shared 2-phenylbenzothiazole framework preserves similar molecular recognition despite substitution of the boronic acid group.

In this study, the aryl boronic acid moiety of BTPB was replaced with iodine-125 through copper-mediated iododeboronation to generate [^125^I]IPBT and with fluorine-18 through copper-mediated nucleophilic radiofluorination to produce [^18^F]FPBT. The structural relationships between BTPB and its radiolabelled analogues are shown in Fig. 1. The radiochemical properties, physicochemical characteristics, cellular distribution, and *in vivo* distribution profiles of the two radiohalogenated analogues were systematically evaluated and compared with selected distribution characteristics of the parent compound where experimentally appropriate. In addition, molecular docking was used to examine whether the shared phenylbenzothiazole scaffold resulted in comparable predicted recognition of AhR. Rather than seeking to establish [^125^I]IPBT or [^18^F]FPBT as a validated pharmacokinetic surrogate for BTPB, the present study aimed to determine how radiohalogen substitution influences the *in vivo* behaviour of BTPB-derived analogues and whether either radiolabelled analogue exhibits selected distribution characteristics relevant to the parent boron agent. In particular, the study explored whether these findings could identify a candidate imaging analogue for further evaluation of BTPB-related intracranial distribution.

**Fig. 1:**
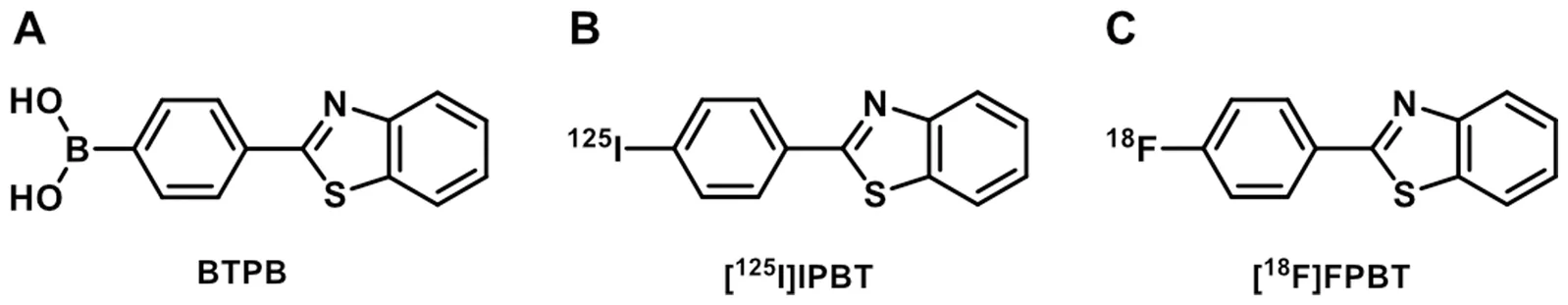
Chemical structures of BTPB (A) and its radiolabelled analogues [^125^I]IPBT (B) and [^18^F]FPBT (C), prepared by copper-mediated iododeboronation and radiofluorination, respectively.

## 2. Experimental

### 2.1. General methods

All chemical reagents and solvents were purchased from Sigma-Aldrich (St Louis, MO, USA), unless otherwise specified, and were used without further purification. Sodium [^125^I]iodide (740 MBq) was obtained from PerkinElmer (Waltham, MA, USA). [^18^F]Fluoride was produced *via* the ^18^O(*p*,*n*)^18^F nuclear reaction using a 16.5 MeV cyclotron (GE Healthcare, Uppsala, Sweden) at the Korea Institute of Radiological and Medical Sciences (KIRAMS). The [^18^F]fluoride was supplied in 1.5 mL [^18^O]water and trapped on a QMA Light SepPak cartridge. BTPB was supplied by FutureChem Co., Ltd (Seoul, Republic of Korea). 2-(4-Fluorophenyl)benzo[*d*]thiazole was purchased from AOBChem (CA, USA). Thin-layer chromatography (TLC) was performed on silica gel 60 *F*_254_ glass plates (20 × 20 cm, 0.25 mm thickness; Merck, Darmstadt, Germany). Nuclear magnetic resonance (NMR) spectra were recorded on a Bruker Avance 600 MHz spectrometer (Bruker Biospin, Rheinstetten, Germany) at 600 MHz (^1^H) and 150 MHz (^13^C). High-resolution mass spectrometry (HRMS) was carried out using a Triple TOF 5600 Plus system (AB Sciex, Framingham, MA, USA) operating in electrospray ionisation (ESI) mode. High-performance liquid chromatography (HPLC) was performed using a Waters system (Milford, MA, USA). For purification and analysis, an XTerra Prep RP18 column (10 µm, 10 mm × 250 mm) and XSelect CSH™ C18 column (5 µm, 4.6 mm × 250 mm) were employed. The HPLC solvent system was as follows: solvent A = H_2_O containing 0.1% trifluoroacetic acid (TFA), solvent B = Acetonitrile (ACN) containing 0.1% TFA; method A: 55% *B* (0–20 min), flow rate = 4 mL min^−1^; method B: 55% *B* (0–5 min); linear gradient to 100% *B* (5–30 min), flow rate = 1 mL min^−1^; method C: 55% *B* (0–5 min); linear gradient to 80% *B* (5–30 min), flow rate = 1 mL min^−1^.

Radiochemical purity and stability were assessed by radio-TLC using aluminium-backed silica gel plates (Merck, Darmstadt, Germany). Radioactivity was measured using a dose calibrator (CRC®-55tR, Capintec, Inc. Ramsey, NJ, USA) in the labelling experiments and using a Wizard2™ gamma counter (γ-counter, PerkinElmer, Waltham, MA, USA) in the biodistribution studies, and radio-TLC profiles were analysed with a Bioscan scanner (Washington, DC, USA).

The human glioblastoma cell line U87MG was obtained from the Korean Cell Line Bank (Seoul, South Korea) and was maintained in Minimum Essential Medium alpha (MEM-α, Corning, NY) supplemented with 10% (v/v) foetal bovine serum (FBS, JR Scientific Inc., Woodland, CA, USA) and 1% (v/v) antibiotics-antimycotics (AA, Gibco, USA). Male BALB/c nude mice (6 weeks old, 20–25 g) were purchased from Japan SLC, Inc. (Hamamatsu, Japan). Boron was quantified in organs using inductively coupled plasma mass spectrometry (ICP-MS; NexION 350D; PerkinElmer, MA, USA). In the orthotopic model, the burr hole was sealed using Kwik-Cast™ (World Precision Instruments, USA) and tumour size was measured using a 9.4 T MRI scanner (Agilent Technologies Inc., Palo Alto, CA, USA). Animal SPECT/CT imaging was performed using the Inveon Multi-Modality micro-SPECT/CT/PET system (Siemens Medical Solutions, Knoxville, USA). Animal PET/CT imaging was performed using a small-animal PET/CT scanner (nanoScan, Mediso Medical Imaging Systems, Budapest, Hungary).

### 2.2. Synthesis of 2-(4-iodophenyl)benzo[*d*]thiazole (IPBT)

IPBT was synthesised *via* copper-mediated iododeboronation of BTPB (Fig. 1A). BTPB (11.8 µmol) and tetrakis(pyridine)copper(ii)triflate (Cu(py)_4_(OTf)_2_, 23.6 µmol) were dissolved in anhydrous methanol (10 mL). Sodium iodide (NaI, 1.8 mg, 11.8 µmol) dissolved in anhydrous methanol (10 mL) was added to the reaction mixture. The reaction was stirred at room temperature for 24 h under ambient atmosphere. Reaction progress was monitored by TLC using a hexane/ethyl acetate (4 : 1, v/v) mobile phase. During the reaction, the solution colour gradually changed from light brown to light blue. After completion, the crude mixture was purified by preparative reversed-phase HPLC (method A), affording IPBT with a retention time (*R*_t_) of 14.79 min and an isolated yield of 80.60% (Fig. S1). ^1^H NMR (600.22 MHz, DMSO-*d*_6_) *δ* 8.17 (d, *J* = 8.0 Hz, 1H), 8.08 (d, *J* = 8.2 Hz, 1H), 7.96 (d, *J* = 8.4 Hz, 2H), 7.88 (d, *J* = 8.5 Hz, 2H), 7.59–7.54 (m, 1H), 7.49 (t, *J* = 8.2 Hz, 1H); ^13^C (150.93 MHz, DMSO-*d*_6_) *δ* 166.39, 153.47, 138.29, 134.46, 132.32, 128.93, 126.83, 125.77, 122.99, 122.42, 98.72; HRMS (ESI) calculated for C_13_H_9_INS [M + H]^+^: 337.9495; found: 337.9497. (Fig. S2–S4).

### 2.3. Optimisation of copper-mediated radioiodination of BTPB

Screening experiments were performed to evaluate the effect of copper catalyst loading, reaction time, and starting activity on radiochemical conversion (RCC, Fig. S5). Precursor-to-catalyst molar ratios ranging from 1 : 0.8 to 1 : 3.5 were evaluated at room temperature for 15 min using 98 nmol (25 µg) of BTPB. Additional experiments were conducted by varying reaction time and initial radioactivity while maintaining constant precursor and catalyst concentrations. RCC was determined by radio-TLC and calculated as the percentage of total radioactivity corresponding to the product peak.

### 2.4. Molecular docking and MM-GBSA calculations

Molecular docking studies were performed using the Schrödinger molecular modelling suite (ver. 14.7; Schrödinger, LLC, New York, NY, USA). The experimentally determined human AhR-ARNT complex structure (ARNT, aryl hydrocarbon receptor nuclear translocator) was obtained from the Protein Data Bank (PDB ID: 8XS9) and prepared using the Protein Preparation Wizard. Hydrogen atoms were added, protonation states were assigned at pH 7.4 ± 2.0, the hydrogen-bonding network was optimised, and the structure was minimised using the OPLS4 force field. BTPB, IPBT, and FPBT were prepared using LigPrep with Epik at pH 7.4 ± 2.0 and minimised using the same force field.

The docking grid was centred on the AhR Per-ARNT-Sim B (PAS-B) binding site defined by the cognate ligand, β-naphthoflavone. Docking was performed using Glide, and the highest-ranked pose based on the docking score was selected for each ligand. The binding energies of the selected complexes were subsequently estimated using Prime MM-GBSA (molecular mechanics with generalised Born and surface area solvation) with the OPLS4 force field. Three-dimensional docking poses and two-dimensional interaction diagrams were generated using Maestro.

### 2.5. Radiolabelling of [^125^I]IPBT

BTPB (0.1 µmol) was reacted with [^125^I]NaI (70–90 MBq) in the presence of Cu(py)_4_(OTf)_2_ (0.3 µmol) in methanol at room temperature for 15 min. For scale-up reactions, BTPB (100 µg) and Cu(py)_4_(OTf)_2_ (824 µg) were dissolved in anhydrous methanol and reacted under identical conditions. The crude reaction mixture was analysed by radio-TLC and purified by reversed-phase HPLC using Method B. The collected radioactive fraction was concentrated using a C18 solid-phase extraction cartridge and formulated in saline containing 10% dimethyl sulfoxide (DMSO).

### 2.6. Production and preparation of [^18^F]fluoride

No-carrier-added [^18^F]fluoride was produced *via* the ^18^O(*p*,*n*)^18^F nuclear reaction using a 16.5 MeV cyclotron at KIRAMS. The irradiated target solution was transferred to a synthesis module, and [^18^F]fluoride was trapped on a QMA light Sep-Pak cartridge. The trapped [^18^F]fluoride was eluted with 550 µL of an aqueous solution containing potassium triflate (KOTf, 5 mg) and K_2_CO_3_ (50 µg). Azeotropic drying was performed by repeated addition and evaporation of ACN (200 µL per cycle) at 100 °C to obtain dried [^18^F]fluoride (3.7–5.55 GBq).

### 2.7. Structural confirmation of 2-(4-fluorophenyl)benzo[*d*]thiazole (FPBT)

The identity of the non-radioactive FPBT reference compound was confirmed by HRMS prior to radiolabelling studies (Fig. S6).

### 2.8. Optimisation of copper-mediated radiofluorination of BTPB

Initial radiofluorination screening was performed using Cu(py)_4_(OTf)_2_ under conditions adapted from the corresponding radioiodination procedure. Subsequent optimisation was conducted using a Cu(OTf)_2_/pyridine system with BTPB as the precursor.

BTPB (4 µmol) was reacted in *N*,*N*-dimethylformamide (DMF), and the amounts of Cu(OTf)_2_ and pyridine were varied to evaluate their effects on RCC. Cu(OTf)_2_ was examined over the range of 5–80 µmol, and pyridine was varied from 75 to 550 µmol. Based on these screening experiments, 5 µmol of Cu(OTf)_2_ and 300 µmol of pyridine were used for subsequent reaction-time and temperature studies.

Reaction times of 5–30 min were evaluated at 110 °C. Reaction temperature was independently examined over the range of 70–150 °C at a fixed reaction time of 15 min. RCC was determined by radio-TLC using silica gel plates with hexane/ethyl acetate (80 : 20, v/v) as the mobile phase. The optimisation conditions and corresponding RCC values are summarised in Fig. S7.

### 2.9. Radiolabelling of [^18^F]FPBT

Dried [^18^F]fluoride was combined with BTPB (4 µmol), Cu(OTf)_2_ (5 µmol), and pyridine (300 µmol) in DMF, and the reaction mixture was heated at 110 °C for 15 min. After completion of the reaction, an aliquot of the crude mixture was analysed by radio-TLC, and the remaining mixture was transferred to the preparative reversed-phase HPLC system for purification. The [^18^F]FPBT-containing fraction was collected, diluted with water, and loaded onto a C18 solid-phase extraction (SPE) cartridge. The product was subsequently eluted with ethanol, concentrated by evaporation of the organic solvent, and formulated in saline containing 10% DMSO. Radiochemical purity was confirmed by analytical radio-HPLC using the corresponding non-radioactive FPBT reference compound for comparison.

Stepwise radioactivity recovery was evaluated across six production runs. All recovery fractions and the overall isolated radiochemical yield (RCY) were calculated using decay-corrected radioactivity values. Pre-HPLC recovery was defined as the activity injected onto the preparative HPLC system relative to the starting activity. HPLC product-fraction recovery was calculated as the activity collected in the [^18^F]FPBT-containing fraction relative to the injected activity, whereas C18 SPE recovery was calculated as the activity recovered after cartridge elution relative to the activity loaded onto the cartridge. Post-evaporation recovery was calculated as the decay-corrected activity remaining after ethanol evaporation relative to the activity recovered in the C18 SPE eluate. The overall isolated RCY was determined from the final isolated product activity relative to the starting activity. The corresponding results are summarised in Table S1 of the SI.

### 2.10. Calibration curves and determination of molar activity

Calibration curves for IPBT and FPBT were generated by analytical HPLC with ultraviolet (UV) detection. Peak areas were plotted against the corresponding amounts of the non-radioactive reference compounds, and linear regression was used for quantitative determination of molar activity (Fig. S8).

### 2.11. *In vitro* radiolabel stability and partition coefficient

The *in vitro* radiolabel stability of [^125^I]IPBT and [^18^F]FPBT was evaluated in normal saline at room temperature and in mouse and human serum at 37 °C. For serum stability studies, [^125^I]IPBT (approximately 4.6 MBq in 85 µL) or [^18^F]FPBT (approximately 8.1 MBq in 30 µL) was mixed with mouse or human serum at a ratio of 1 : 5 (v/v). Samples containing [^125^I]IPBT were collected at 0, 1, 4, 7, and 12 h, whereas samples containing [^18^F]FPBT were collected at 10, 30, 60, and 120 min. Radiolabel stability was determined by radio-TLC.

The partition coefficients (log *P*) of [^125^I]IPBT and [^18^F]FPBT were determined using an *n*-octanol/water partitioning system (1 : 1, v/v). After vigorous mixing and centrifugation, aliquots of the organic and aqueous phases were collected and their radioactivities were measured using a γ-counter. Log *P* was calculated from the ratio of radioactivity in the *n*-octanol phase to that in the aqueous phase.

### 2.12. Cell culture, cellular uptake, and subcellular distribution

U87MG cells were cultured at 37 °C in a humidified atmosphere containing 5% CO_2_. Cellular uptake was evaluated after incubation for 30, 60 and 120 min for [^125^I]IPBT and 10 and 30 min for [^18^F]FPBT. Briefly, 500 µL of serum-free medium containing [^125^I]IPBT (0.02–0.03 MBq) or [^18^F]FPBT (0.09–0.11 MBq) was added to vented culture tubes containing 1 × 10^6^ cells suspended in 500 µL of serum-free medium. After incubation, the cells were washed three times with ice-cold phosphate-buffered saline (PBS), and cell-associated radioactivity was quantified using a γ-counter. Cellular uptake was expressed as the percentage of added radioactivity per 1 × 10^6^ cells. Data are presented as mean ± SD from three independent experiments (*n* = 3, Fig. S9).

Subcellular fractionation was performed at the corresponding incubation times using a subcellular protein fractionation kit (Thermo Fisher Scientific, Waltham, MA, USA) according to the manufacturer's instructions. Cell-associated radioactivity in the cytosolic, membrane, nuclear, and cytoskeletal fractions was measured using a γ-counter and expressed as a percentage of the total cell-associated radioactivity.

### 2.13. Preparation of U87MG tumour models

All animal procedures were performed in accordance with the guidelines of the Institutional Animal Care and Use Committee (IACUC) of the Korea Institute of Radiological and Medical Sciences and were approved under protocol no. KIRAMS 2024-0037.

Male BALB/c nude mice (6 weeks old, 20–25 g) were housed under controlled environmental conditions at 23 °C with free access to food and water. For the subcutaneous xenograft model, U87MG cells (1 × 10^6^ cells) suspended in 100 µL of serum-free Dulbecco's modified Eagle's medium (DMEM) were injected subcutaneously into the right thigh. When tumour volumes reached approximately 100 mm^3^, animals were randomly assigned to PET/CT imaging, SPECT/CT imaging, or biodistribution studies.

For the orthotopic glioblastoma model, mice were anaesthetised with 2% isoflurane in oxygen and secured in a stereotactic frame. A burr hole was created 3 mm lateral and 1 mm anterior to the bregma, and U87MG cells (1 × 10^6^ cells in 5 µL serum-free DMEM) were injected at a rate of 1 µL min^−1^ using a Hamilton microsyringe. After injection, the needle was maintained in position for 1 min before being slowly withdrawn to minimise reflux. The burr hole was sealed with Kwik-Cast (World Precision Instruments, USA), and the incision was closed by suturing. Tumour progression was monitored at 2-week intervals using a 9.4 T MRI, and animals were used for biodistribution studies 4 weeks after implantation.

### 2.14. Biodistribution studies

For radiotracer biodistribution studies, [^125^I]IPBT (0.07–0.09 MBq) was administered intravenously *via* the tail vein to mice bearing subcutaneous U87MG tumours (*n* = 4). [^18^F]FPBT (5.29–7.55 MBq) was administered intravenously to mice bearing either subcutaneous (*n* = 4) or orthotopic (*n* = 2) U87MG tumours. Animals were euthanised at the designated time points after injection. Blood, tumour, brain, kidney, liver, muscle, lung, intestine, stomach, spleen, heart, and tail samples were collected and weighed, and their radioactivity was measured using a γ-counter. Tissue uptake was expressed as the percentage of injected dose per gram of tissue (% ID/g).

For quantitative boron biodistribution, mice bearing subcutaneous or orthotopic U87MG tumours received BTPB (250 mg kg^−1^) intravenously in 100 µL of normal saline containing 10% DMSO and 13% tween 80. At 0.5, 1, 2, and 4 h after administration (*n* = 3–4), tissues were collected, weighed, and digested in 70% nitric acid at 100 °C for 1 h. The digested samples were diluted with deionised water, and boron concentrations were determined by ICP-MS and were expressed as µg B g^−1^ tissue.

### 2.15. SPECT/CT imaging

SPECT/CT imaging was performed using an Inveon Multi-Modality micro-SPECT/CT/PET system. Mice bearing subcutaneous U87MG tumours were anaesthetised with 2.5% isoflurane in oxygen, and [^125^I]IPBT (7.44–8.62 MBq) formulated in saline containing 10% DMSO was administered intravenously *via* the tail vein. Static SPECT acquisitions were performed for 25 min at 30, 60, 90, 300, and 420 min post-injection. Projection data were reconstructed using the manufacturer's reconstruction algorithm. Regions of interest (ROIs) were manually delineated using Inveon Research Workplace, and standardised uptake values (SUVs) were calculated for quantitative analysis.

### 2.16. PET/CT imaging

PET/CT imaging was performed using a nanoScan PET/CT system in mice bearing subcutaneous U87MG tumours. Animals were anaesthetised with 2.5% isoflurane in oxygen, and [^18^F]FPBT (4.77 MBq) formulated in saline containing 10% DMSO was administered intravenously *via* the tail vein over 5 min using a syringe pump. Dynamic PET data were acquired for 60 min using six sequential acquisition frames.

PET images were reconstructed using a three-dimensional ordered-subset expectation maximisation (3D-OSEM) algorithm with four iterations and six subsets. The energy window was 400–600 keV. Immediately after PET acquisition, CT images were acquired at 50 kVp and 0.16 mAs for attenuation correction and anatomical co-registration. ROIs were manually delineated, and SUVs were calculated for quantitative analysis.

## 3. Results and discussion

### 3.1. Radiosynthesis of the radiolabelled BTPB analogues

To generate radiolabelled analogues of BTPB for comparative biodistribution and pharmacokinetic evaluation, radiohalogenation strategies using BTPB as a common precursor were investigated. The aryl boronic acid moiety of BTPB provides a versatile synthetic handle for the radiohalogen incorporation through transition-metal-mediated radiofluorination and iododeboronation reactions.^17–22^ The radiosynthetic routes to [^125^I]IPBT and [^18^F]FPBT are summarised in Scheme 1.

**Scheme 1:**
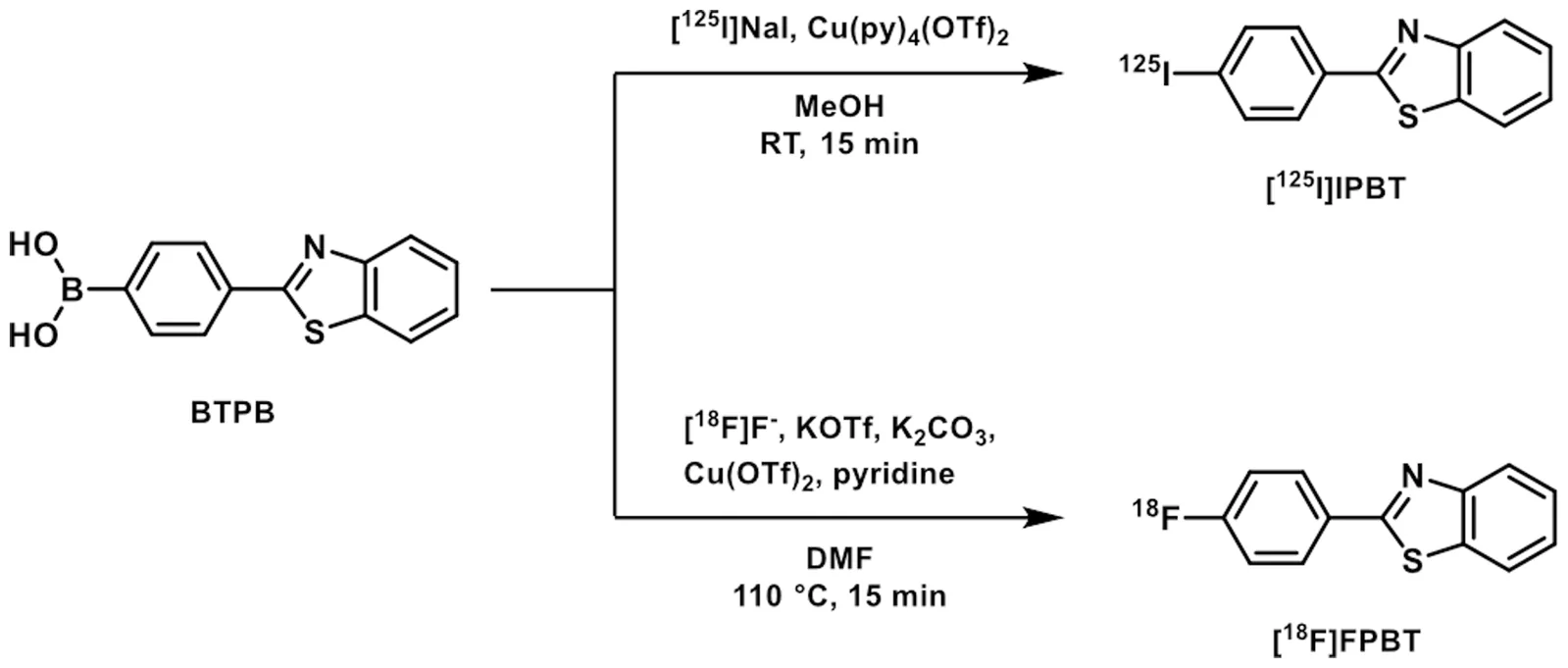
Radiosynthetic routes to the radiolabelled BTPB analogues [^125^I]IPBT and [^18^F]FPBT from the common boronic acid precursor BTPB. [^125^I]IPBT was prepared by copper-mediated iododeboronation with [^125^I]NaI, whereas [^18^F]FPBT was synthesised by copper-mediated nucleophilic radiofluorination with [^18^F]fluoride.

The aryl boronic acid functionality of BTPB was first exploited for copper-mediated radioiodination.^19–21^ Reaction time and copper catalyst loading were screened at room temperature using 0.1 µmol of BTPB, and RCC was monitored by radio-TLC. Although the highest RCC was observed after 30 min, a reaction time of 15 min was selected for subsequent radiosynthesis because a high conversion had already been achieved at this time point. RCC was also strongly dependent on copper loading, with efficient conversion requiring at least a stoichiometric amount of Cu(py)_4_(OTf)_2_ relative to the precursor (Fig. S5). Under the selected conditions, the crude reaction mixture was purified by preparative radio-HPLC to afford [^125^I]IPBT with an RCY of 68.76 ± 1.16% (*n* = 3). The purified product was subsequently formulated using a C18 Sep-Pak cartridge in saline containing 10% DMSO. Analytical radio-HPLC confirmed a radiochemical purity of >95%. The radioactive product co-eluted with the corresponding non-radioactive IPBT reference compound, with retention times (*R*_t_) of 24.282 and 23.900 min, respectively, using Method B (Fig. 2A). The overall isolated RCY was 28.39 ± 5.12% (*n* = 3), and the molar activity at the end of synthesis was 48.9 GBq µmol^−1^, as determined using an analytical calibration curve (Fig. S8A).

**Fig. 2:**
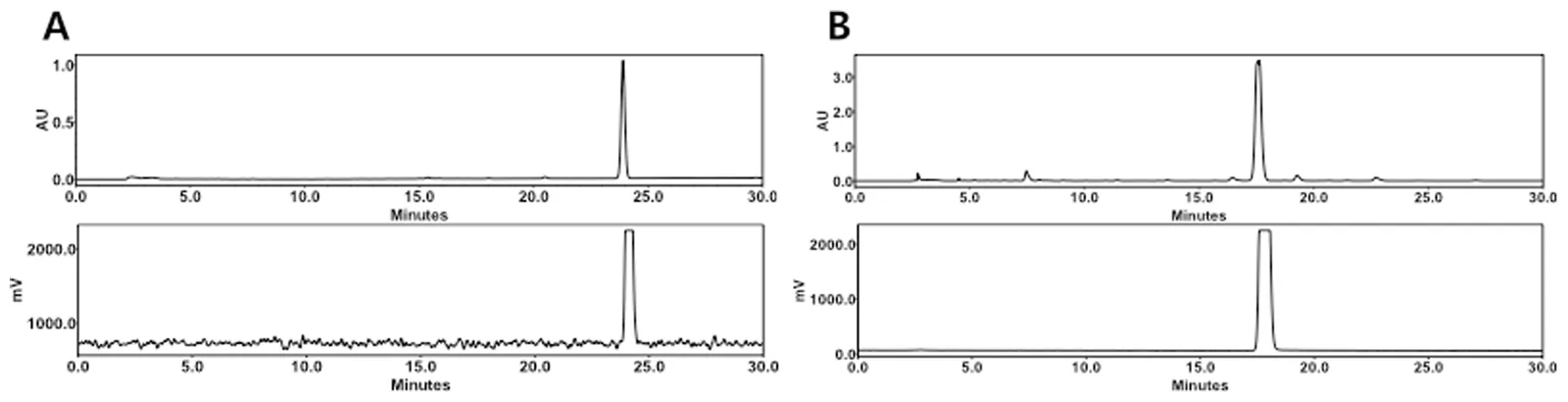
Analytical radio-high-performance liquid chromatography (radio-HPLC) characterisation of [^125^I]IPBT and [^18^F]FPBT. Radiochromatograms (top) and ultraviolet (UV) chromatograms of the corresponding non-radioactive reference compounds (bottom) demonstrated co-elution under identical chromatographic conditions. Panel (A) shows [^125^I]IPBT and IPBT; panel (B) shows [^18^F]FPBT and FPBT.

For [^18^F]FPBT radiosynthesis, Cu(py)_4_(OTf)_2_-based conditions initially adapted from the corresponding radioiodination procedure did not produce detectable [^18^F]FPBT under the conditions investigated. The radiofluorination system was therefore modified to employ KOTf/K_2_CO_3_ for [^18^F]fluoride elution and Cu(OTf)_2_ with free pyridine for copper-mediated radiofluorination.^17,18,22^

The effects of Cu(OTf)_2_ and pyridine loading were first examined using BTPB (4 µmol) in DMF (Fig. S7A). At a fixed pyridine amount of 300 µmol, the highest RCC was obtained using 5 µmol of Cu(OTf)_2_, whereas increasing the copper loading did not further improve conversion. Pyridine loading was subsequently varied at 5 µmol of Cu(OTf)_2_, and 300 µmol was selected for further optimisation. Using these reagent amounts, reaction time and temperature were systematically investigated (Fig. S7B). At 110 °C, RCC increased from 74.17% at 5 min to 77.61% at 10 min and reached 80.07% and 80.21% at 15 and 20 min, respectively. Extension of the reaction to 25 or 30 min did not provide further improvement. In an independent temperature-screening experiment performed at a fixed reaction time of 15 min, RCC increased from 46.53% at 70 °C to 58.35% at 90 °C and 76.76% at 110 °C. Increasing the temperature to 120 or 130 °C provided no additional improvement, whereas RCC decreased at 150 °C. Based on these results, 110 °C for 15 min was selected as the final reaction condition because it provided near-maximal RCC while maintaining a relatively short reaction time.

Despite the relatively high RCC achieved during reaction optimisation, the overall isolated RCY of [^18^F]FPBT remained modest. To investigate the factors contributing to this discrepancy, stepwise radioactivity recovery was evaluated across six production runs (Table S1). The mean pre-HPLC recovery was 57.53 ± 14.09%, indicating a substantial reduction in recoverable activity before preparative HPLC injection. Residual radioactivity in the reaction vessel and incomplete recovery during transfer of the crude reaction mixture were consistently observed across the analysed runs, suggesting that pre-purification handling contributed to activity loss. However, pre-HPLC recovery represents cumulative activity recovery up to the HPLC injection stage and therefore does not exclusively reflect transfer efficiency.

Following preparative HPLC injection, 14.15 ± 2.89% of the injected activity was recovered in the [^18^F]FPBT-containing product fraction. This value represents product-fraction recovery rather than chromatographic recovery alone, because the crude reaction mixture contained unreacted [^18^F]fluoride and other radioactive species that were separated from the desired product during purification. In contrast, C18 solid-phase extraction afforded a recovery of 92.49 ± 2.77%, indicating that C18 trapping and elution were comparatively efficient. However, subsequent ethanol evaporation resulted in a further reduction in recovered radioactivity, with a mean post-evaporation recovery of 56.92 ± 17.64%. The overall decay-corrected isolated RCY was 3.10 ± 1.35% (*n* = 6). These findings indicate that incomplete recovery and transfer of the crude reaction mixture, together with limited recovery of product-associated activity during preparative isolation, contributed to the modest isolated yield. Additional losses during subsequent handling, concentration, and formulation may also have contributed, although their individual contributions could not be fully quantified. Further optimisation of these processes is therefore required to improve the overall radiosynthetic efficiency.

Analytical radio-HPLC confirmed a radiochemical purity of >99%, and the radioactive product co-eluted with the corresponding non-radioactive FPBT reference compound, with *R*_t_ values of 17.592 and 17.615 min, respectively, using Method C (Fig. 2B). The molar activity of [^18^F]FPBT at the end of synthesis was 6.84 GBq µmol^−1^ (Fig. S8B).

The non-radioactive FPBT scaffold and its radiosynthesis have previously been reported in the context of amyloid-targeted PET imaging.^27^ In the present study, however, [^18^F]FPBT was investigated as a radiofluorinated analogue of the BNCT agent BTPB to assess whether it shares selected distribution characteristics with the parent boron compound rather than its amyloid-targeting properties. Both radiosynthetic strategies replace the aryl boronic acid moiety of BTPB with the corresponding radiohalogen, yielding structurally related analogues suitable for comparative pharmacokinetic evaluation.

### 3.2. Physicochemical characterisation

Because the *in vivo* behaviour of radiolabelled compounds can be strongly influenced by their physicochemical properties, radiolabel stability and lipophilicity were evaluated before *in vivo* pharmacokinetic studies. The *in vitro* radiolabel stability of [^125^I]IPBT and [^18^F]FPBT was evaluated in normal saline at room temperature and in mouse and human serum at 37 °C (Fig. 3). Both radiolabelled analogues showed generally high stability over the investigated incubation periods, with the majority of radioactivity remaining associated with the parent radiolabelled species. [^18^F]FPBT showed slightly lower serum stability, with approximately 85% of the parent compound remaining, but retained sufficient stability for subsequent *in vivo* evaluation.

**Fig. 3:**
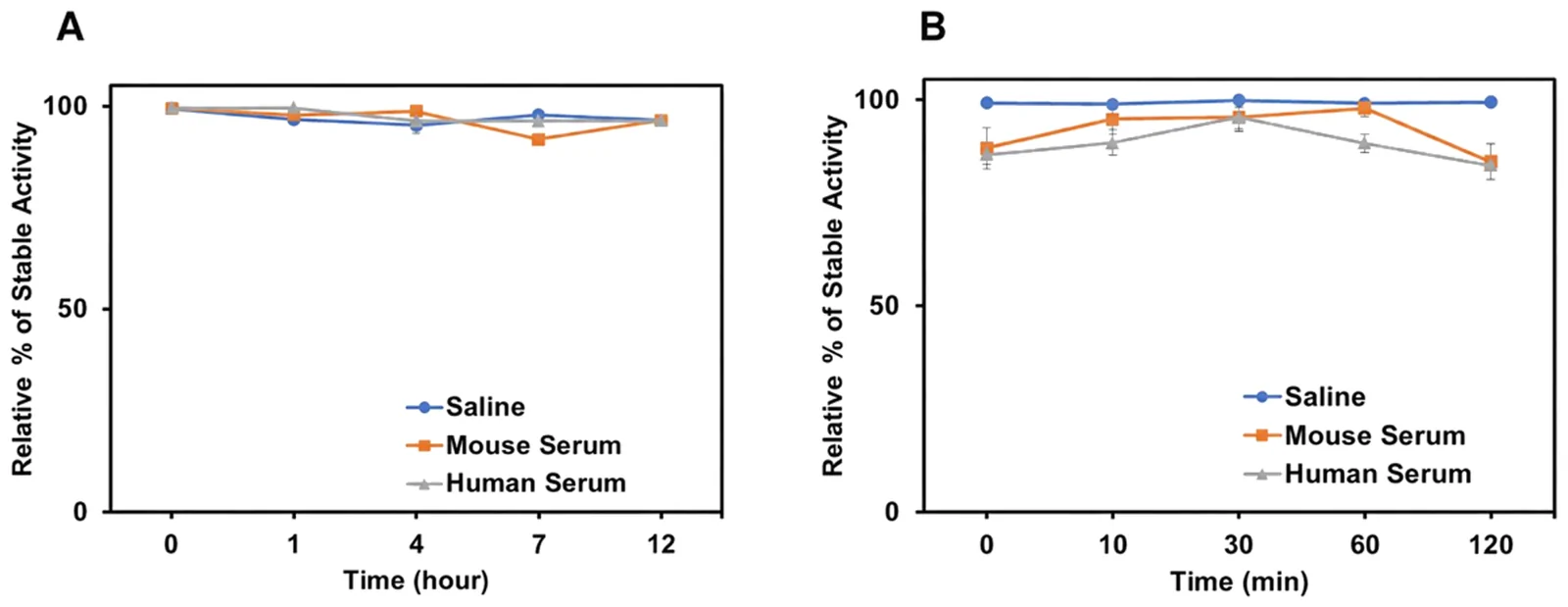
*In vitro* radiolabel stability of (A) [^125^I]IPBT and (B) [^18^F]FPBT after incubation in normal saline at room temperature and in mouse and human serum at 37 °C. Data are presented as mean ± SD (*n* = 3).

The lipophilicities of [^125^I]IPBT and [^18^F]FPBT were evaluated using an *n*-octanol/water partitioning system. The experimentally determined log *P* values were 1.71 and 1.69, respectively, indicating similar moderate lipophilicity. Topological polar surface area (tPSA) values for IPBT and FPBT were calculated according to the method described by Ertl *et al.*^28^ These physicochemical descriptors were further evaluated using the BOILED-egg model implemented in SwissADME to estimate BBB permeability (Fig. S10).^29,30^ Both compounds were predicted to fall within the BBB-permeable region of the model, supporting subsequent investigation of their intracranial distribution.

Importantly, despite their nearly identical experimental log *P* values and similar predicted BBB permeability, [^125^I]IPBT and [^18^F]FPBT subsequently exhibited distinct *in vivo* distribution profiles. These findings suggest that lipophilicity alone is insufficient to account for the pharmacokinetic differences between the two radiohalogenated analogues.

### 3.3. Molecular docking analysis

To investigate whether the radiolabelled analogues retain molecular recognition characteristics similar to those of BTPB, molecular docking studies were performed using experimentally determined AhR-ARNT complex structure (PDB ID: 8XS9).^31^ The ligand-binding site was initially characterised on the basis of the binding mode of β-naphthoflavone.^31,32^ Structural analysis indicated that the ligand occupies a predominantly hydrophobic pocket containing several aromatic residues, providing a favourable environment for aromatic ligands. These structural features are relevant to BTPB, IPBT, and FPBT, which share a 2-phenylbenzothiazole scaffold capable of forming π–π and hydrophobic interactions within the binding pocket.

Molecular docking showed that BTPB, IPBT, and FPBT could be accommodated within the AhR PAS-B ligand-binding domain, with predicted docking scores of −9.072, −8.651, and −9.112 kcal mol^−1^, respectively (Fig. 4). The corresponding three-dimensional docking poses and two-dimensional interaction diagrams indicated that the aromatic rings were positioned near His289 and Phe293 or Phe349, supporting favourable π–π and hydrophobic interactions. FPBT and IPBT exhibited similar binding patterns, including an additional polar interaction between the benzothiazole nitrogen and Gln381. BTPB adopted a slightly different local orientation, with the boronic acid group directed towards the polar region near Thr287 and His289. MM-GBSA calculations yielded predicted binding energies of −55.72, −64.52, and −58.98 kcal mol^−1^ for BTPB, IPBT, and FPBT, respectively. Despite differences in the calculated binding energies and local ligand-residue interactions, BTPB, IPBT, and FPBT retained comparable orientations of the shared 2-phenylbenzothiazole core. This similarity suggests that the common aromatic scaffold may contribute substantially to molecular recognition within the AhR binding pocket, whereas the boronic acid, fluorine, and iodine substituents may modulate local interactions with surrounding residues. Thus, radiohalogen substitution appears to preserve the overall predicted AhR-binding orientation of the parent scaffold.

**Fig. 4:**
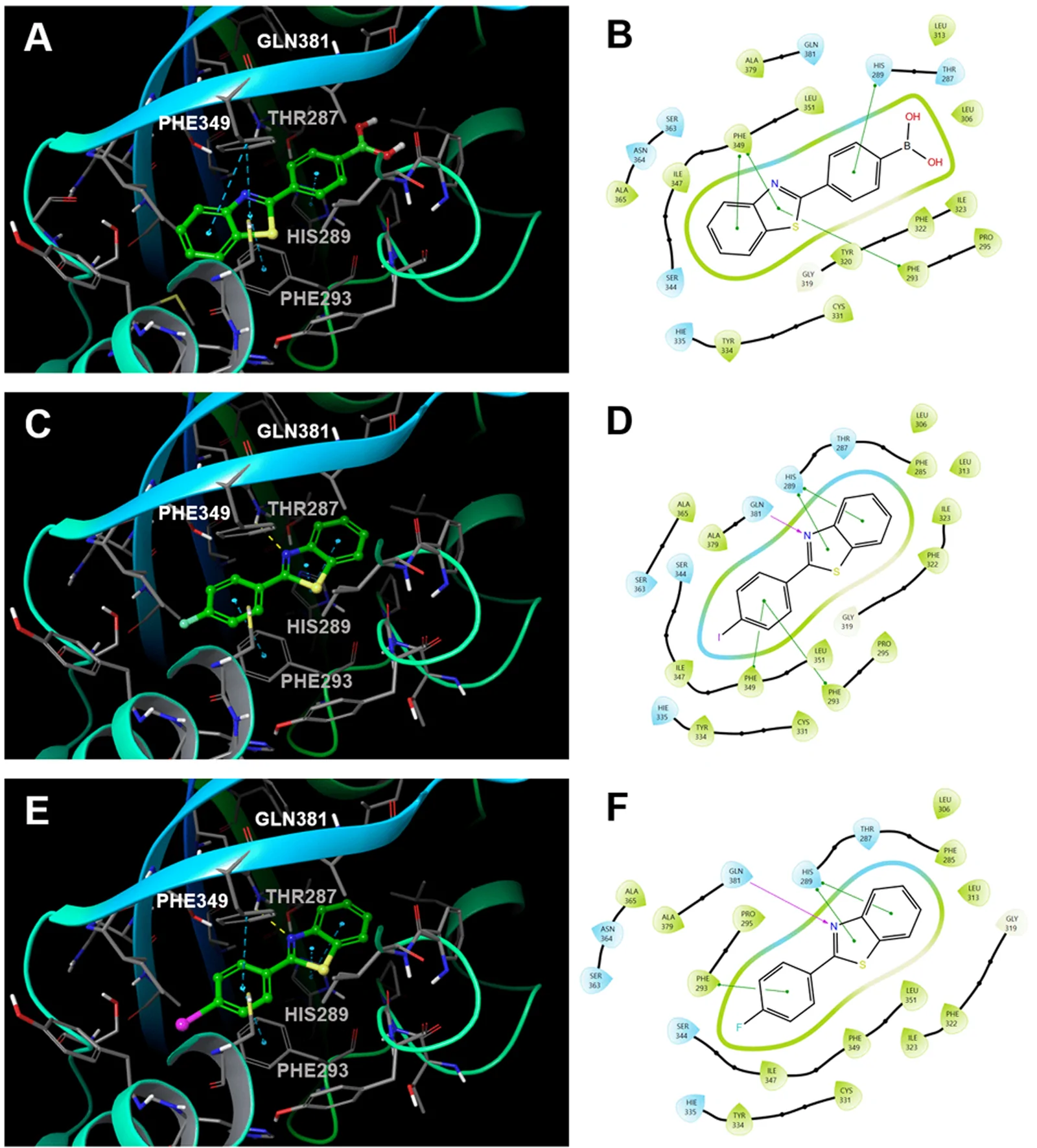
Predicted binding modes and ligand-residue interactions of BTPB, IPBT, and FPBT within the aryl hydrocarbon receptor (AhR) Per-ARNT-Sim B (PAS-B) ligand-binding domain. Three-dimensional docking poses and corresponding two-dimensional interaction diagrams are shown for BTPB (A and B), IPBT (C and D), and FPBT (E and F), respectively. Key residues within the ligand-binding pocket, including Thr287, His289, Phe293, Phe349, and Gln381, are labelled.

Nevertheless, the distinct pharmacokinetic profiles observed for BTPB, [^125^I]IPBT, and [^18^F]FPBT indicate that AhR binding alone is unlikely to account for their *in vivo* behaviour, which may additionally be influenced by physicochemical properties, metabolic stability, and organ-specific clearance. Previous radiopharmaceutical studies have shown that radiohalogen substitution can influence metabolic stability and tissue pharmacokinetics, particularly through *in vivo* dehalogenation and halogen-dependent differences in distribution.^23,24^ In peptide-based radiopharmaceuticals, renal handling is an important determinant of whole-body distribution, and transporter-mediated renal processes can further influence pharmacokinetic behaviour.^33,34^ Labelling strategy and the metabolic stability of radioiodinated compounds are also important considerations in determining their *in vivo* behaviour.^35,36^ For compounds intended for intracranial applications, BBB permeability and carrier-mediated transport represent additional determinants of brain exposure.^37–39^ These considerations are particularly relevant to BNCT theranostic development, in which an imaging agent is expected to reflect relevant distribution characteristics of the corresponding boron-containing compound.

### 3.4. Cellular uptake and subcellular distribution

The cellular uptake and subcellular distribution of [^125^I]IPBT and [^18^F]FPBT were investigated in U87MG cells (Fig. S9). Subcellular fractionation was performed, and the results were interpreted following established approaches for evaluating the intracellular distribution of radiopharmaceuticals and small molecules.^40,41^ Cellular uptake increased with incubation time for both radiolabelled analogues (Fig. S9A and B); however, differences were observed in their intracellular distribution profiles. [^18^F]FPBT showed a greater proportion of cell-associated radioactivity in the nuclear fraction, particularly at the early 10 min time point, whereas [^125^I]IPBT was predominantly associated with the membrane and cytosolic fractions (Fig. S9C and D). These results demonstrate that replacement of the boronic acid group with different radiohalogens can be accompanied by differences in intracellular distribution despite similar bulk lipophilicity. However, the biological significance of these differences remains unclear, and a direct relationship between subcellular localisation and the subsequent *in vivo* pharmacokinetic profiles cannot be established from the present data.

### 3.5. Comparative evaluation of temporal distribution using AUC-based organ-to-blood ratios

Biodistribution studies were performed in U87MG tumour-bearing mice to compare the temporal distribution profiles of [^125^I]IPBT and [^18^F]FPBT with that of the parent compound BTPB (Fig. S11). Cumulative tissue exposure relative to systemic circulation was assessed using organ-to-blood area-under-the-curve (AUC) ratios. Because the datasets differed in administered mass and analytical methodology, these normalised AUC ratios were used to compare temporal distribution patterns rather than absolute tissue concentrations among the three compounds. BTPB exhibited the highest brain-to-blood AUC ratio during the earliest interval (0.5–1 h), followed by a progressive decline (Fig. 5A). [^18^F]FPBT showed a similar temporal pattern, although its brain-to-blood ratios remained lower than those of BTPB. By contrast, [^125^I]IPBT exhibited lower brain-to-blood ratios throughout the investigated intervals and showed a different temporal brain distribution pattern from BTPB. A similar distinction was observed in tumour tissue (Fig. 5B). The tumour-to-blood AUC ratio of BTPB reached its maximum during the 1–2 h interval, indicating delayed tumour enrichment relative to systemic circulation. [^18^F]FPBT exhibited the same temporal tendency, with its highest tumour-to-blood ratio occurring during the 1–2 h interval followed by a decrease during the 2–4 h interval. By contrast, [^125^I]IPBT showed its highest tumour-to-blood ratio during the earliest interval (0.5–1 h), followed by a progressive decrease.

**Fig. 5:**
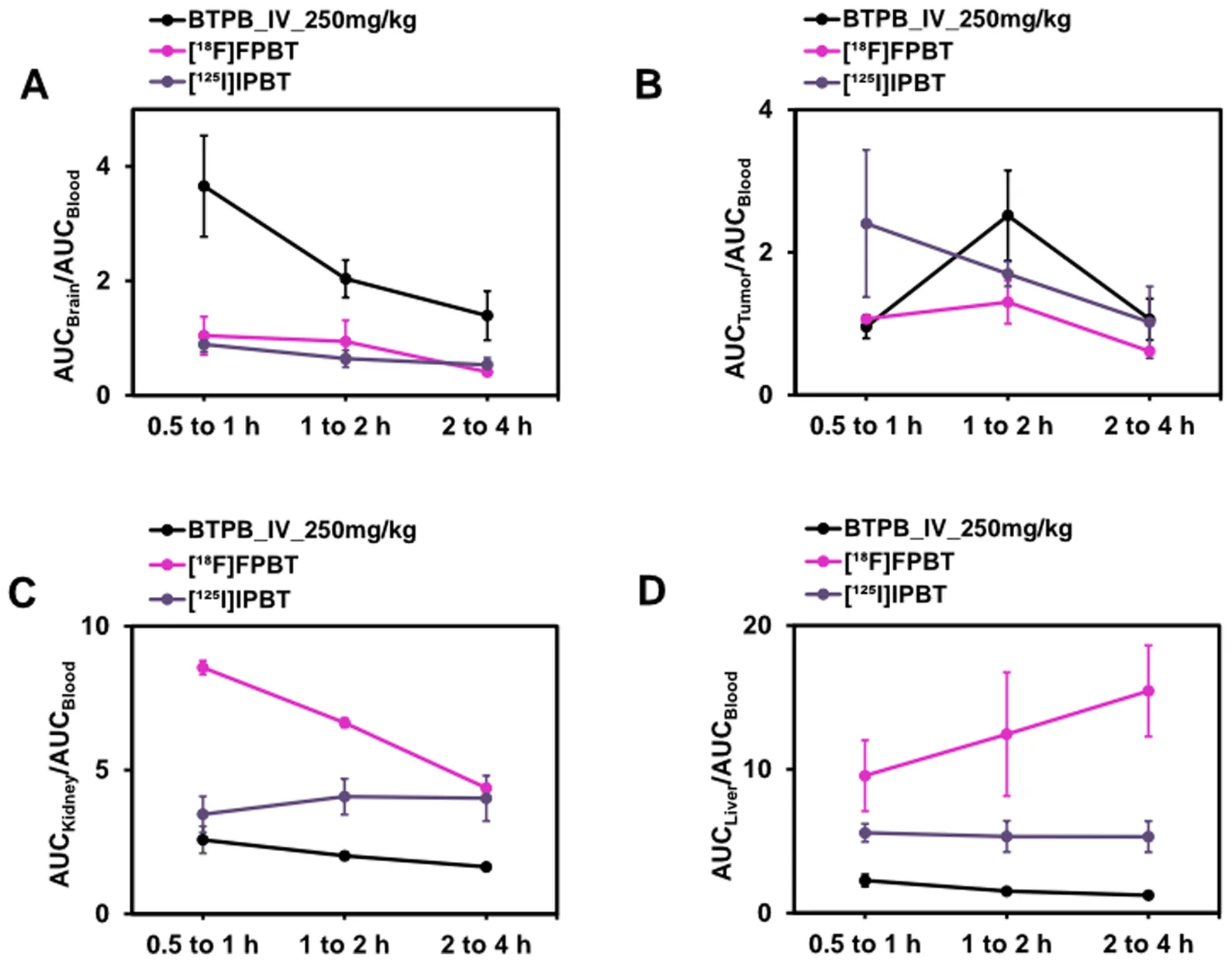
Time-dependent organ-to-blood area-under-the-curve (AUC) ratios of BTPB and its radiolabelled analogues after intravenous administration. (A) Brain-to-blood, (B) tumour-to-blood, (C) kidney-to-blood, and (D) liver-to-blood AUC ratios calculated over the 0.5–1, 1–2, and 2–4 h intervals after administration. Data are presented as mean ± SD.

Differences were also observed in the major clearance organs. Kidney-to-blood AUC ratios were consistently higher for [^18^F]FPBT than for BTPB throughout the investigated intervals, whereas [^125^I]IPBT showed intermediate values (Fig. 5C). In the liver, [^18^F]FPBT exhibited increasing liver-to-blood AUC ratios over time, [^125^I]IPBT showed relatively stable intermediate values, and BTPB exhibited comparatively low hepatic exposure (Fig. 5D). Notably, the absolute biodistribution data showed greater tumour retention of [^125^I]IPBT than of [^18^F]FPBT at later time points (Fig. S11). However, this prolonged retention was not accompanied by the temporal tumour-to-blood distribution pattern observed for BTPB. BTPB was characterised by delayed tumour enrichment relative to blood, with the highest tumour-to-blood exposure occurring during the 1–2 h interval; a similar timing of maximal relative tumour exposure was observed for [^18^F]FPBT.

These findings demonstrate that absolute tissue retention alone does not necessarily reflect similarity in temporal distribution to the parent compound and highlight the value of comparing normalised temporal distribution patterns. Within the experimental time window, [^18^F]FPBT showed selected temporal distribution features that were qualitatively consistent with those observed for BTPB, particularly in brain and tumour tissue, whereas [^125^I]IPBT exhibited distinct temporal patterns. Nevertheless, differences in renal and hepatic distribution, together with differences in administered mass and analytical methodology, preclude interpretation of these observations as evidence of pharmacokinetic equivalence between [^18^F]FPBT and BTPB. The importance of such comparative distribution analysis is also reflected in the development of structurally matched diagnostic and therapeutic agents for BNCT, for which correspondence between imaging and boron distribution is a central consideration.^42,43^ Ultimately, reliable translation of biodistribution information into BNCT treatment planning requires accurate spatial and temporal characterisation of boron distribution and appropriate treatment-planning methodologies.^44,45^

### 3.6. Imaging-based comparison of *in vivo* distribution

Small-animal SPECT/CT and PET/CT imaging was performed to visualise the *in vivo* distribution of [^125^I]IPBT and [^18^F]FPBT, respectively (Fig. 6). SPECT/CT imaging of [^125^I]IPBT showed predominant radioactivity in the gastrointestinal region and thyroid, with limited brain uptake throughout the imaging period (Fig. 6A). Quantitative SUV analysis showed that hepatic uptake increased during the early imaging period, reaching a maximum at approximately 90 min before declining, whereas renal uptake remained relatively low at earlier time points but increased at the final imaging time point (420 min) (Fig. 6B). The increasing thyroid-associated activity, together with the altered whole-body distribution, was consistent with possible *in vivo* deiodination of the radioiodinated analogue.^23^ The imaging profile therefore differed substantially from the intracranial distribution characteristics previously reported for BTPB. By contrast, PET/CT imaging of [^18^F]FPBT demonstrated prominent early brain uptake during the initial 0–10 min acquisition interval (Fig. 6C). The corresponding SUV analysis showed that brain uptake was highest during this initial interval and subsequently declined, while hepatic and renal uptake generally decreased from their early peak values, with some fluctuations in renal activity at later time points (Fig. 6D). Bone-associated radioactivity remained low during the imaging period, providing no clear evidence of extensive *in vivo* defluorination. The PET imaging findings were broadly consistent with the corresponding *ex vivo* biodistribution data. Taken together, the imaging studies support the biodistribution results, showing marked differences in the *in vivo* behaviour of the two radiohalogenated analogues. In particular, [^18^F]FPBT showed early brain uptake, a qualitative distribution characteristic also observed for BTPB, whereas [^125^I]IPBT exhibited limited brain uptake, although substantial quantitative differences in organ distribution remained.

**Fig. 6:**
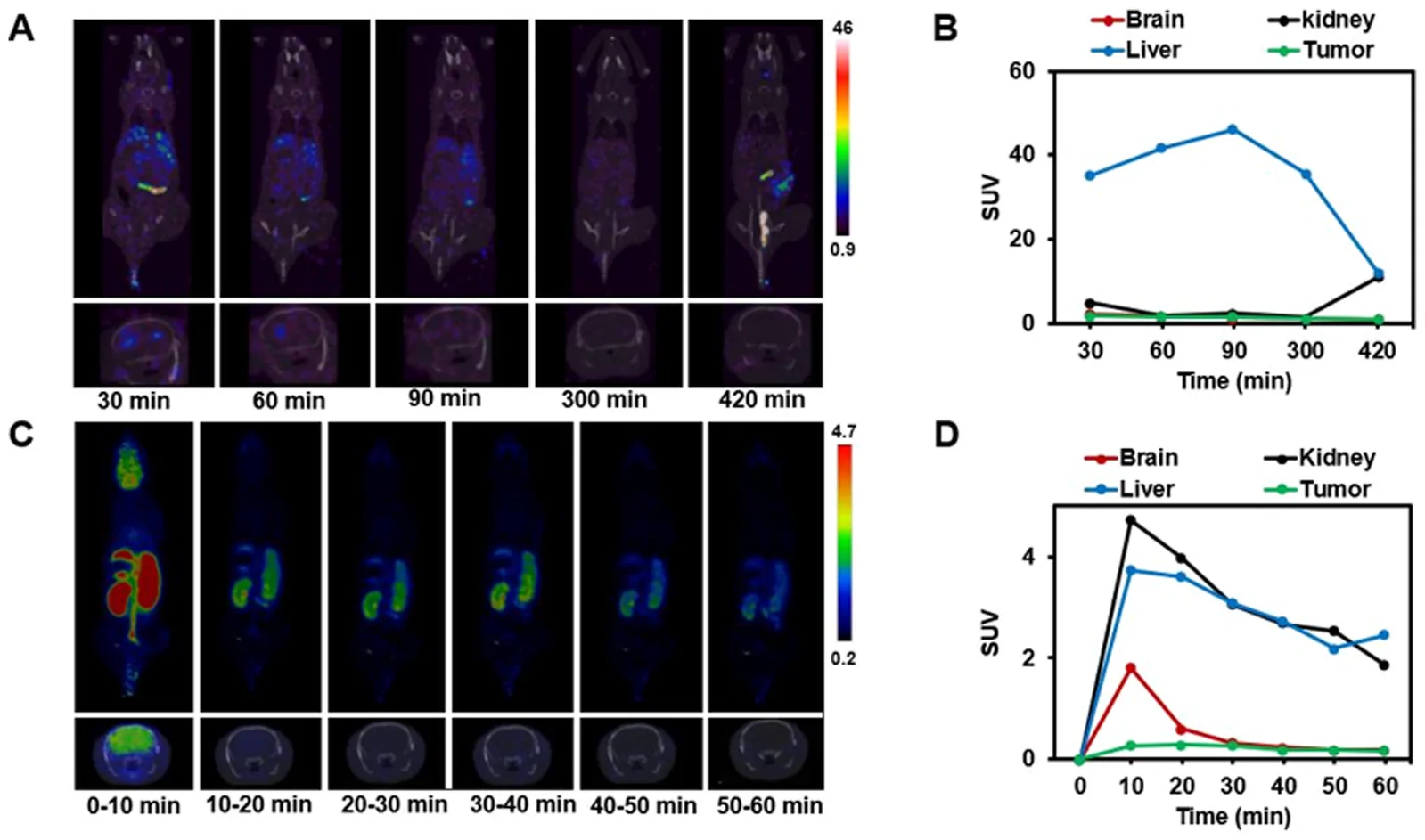
Single-photon emission computed tomography/computed tomography (SPECT/CT) and positron emission tomography/computed tomography (PET/CT) imaging of radiolabelled BTPB analogues in U87MG tumour-bearing mice. (A) Representative SPECT/CT images of [^125^I]IPBT acquired at 30, 60, 90, 300, and 420 min after intravenous administration. (B) Standardised uptake value (SUV) time-activity curves for brain, liver, kidney, and tumour derived from SPECT imaging. (C) Representative dynamic PET/CT images of [^18^F]FPBT acquired during the first 60 min after intravenous administration. (D) SUV time-activity curves for brain, liver, kidney, and tumour derived from PET imaging.

### 3.7. Evaluation in an orthotopic glioblastoma model

Because BTPB was developed as a boron agent with favourable intracranial distribution characteristics, the intracranial distribution of [^18^F]FPBT was further examined in an orthotopic U87MG glioblastoma model and qualitatively compared with that of BTPB. [^18^F]FPBT distribution was quantified by *ex vivo* γ-counting of dissected tissues at 10 and 30 min after administration, whereas boron concentrations after administration of non-radioactive BTPB were determined by ICP-MS at 2 and 4 h. [^18^F]FPBT showed high early uptake in normal brain tissue, reaching 7.23 ± 1.34% ID/g at 10 min, followed by a marked decrease to 1.63 ± 0.28% ID/g at 30 min (Fig. 7A). Tumour-associated radioactivity decreased to a lesser extent over the same interval, from 2.36 ± 0.37 to 1.26 ± 0.30% ID/g. Consequently, the tumour-to-brain (T/Br) ratio increased from 0.33 at 10 min to 0.77 at 30 min, indicating a greater reduction in radioactivity in normal brain than in tumour tissue during this early observation period. In the separate BTPB experiment, a qualitatively similar directional change was observed over a later time interval (Fig. 7B). Between 2 and 4 h after BTPB administration, the boron concentration in normal brain decreased more markedly than that in tumour tissue, likewise resulting in an increase in the T/Br ratio.

**Fig. 7:**
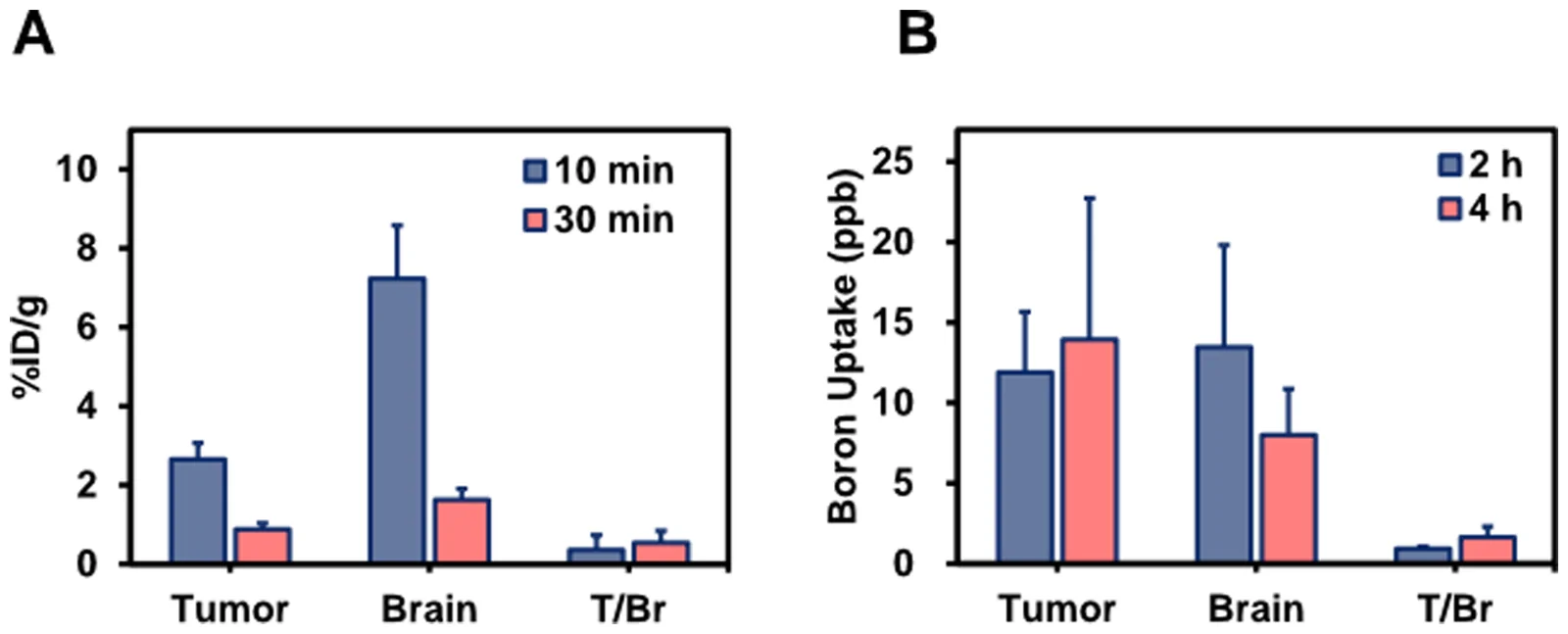
Comparison of tumour, normal-brain, and tumour-to-brain (T/Br) ratios in the orthotopic U87MG glioblastoma model. (A) *Ex vivo* biodistribution of [^18^F]FPBT determined by γ-counting at 10 and 30 min after intravenous administration. (B) Boron concentration after BTPB administration determined by inductively coupled plasma mass spectrometry (ICP-MS) at 2 and 4 h after intravenous administration. Data are presented as mean ± SD.

The purpose of this comparison was not to establish pharmacokinetic equivalence between [^18^F]FPBT administered at a tracer dose and BTPB administered at a pharmacological dose, but rather to examine whether a radiolabelled BTPB analogue could exhibit selected intracranial distribution features relevant to imaging the parent boron agent. Despite the substantially different administered masses and observation windows, both datasets showed the same qualitative direction of temporal change: brain exposure decreased over time, with a greater reduction in normal brain than in tumour tissue, resulting in increased tumour-to-brain contrast within their respective observation periods. These findings should therefore be interpreted as a qualitative similarity in temporal behaviour rather than evidence of pharmacokinetic correspondence between [^18^F]FPBT and BTPB.

From the perspective of developing an imaging analogue for BTPB, this qualitative behaviour is relevant because a useful tracer would ideally demonstrate early brain access and subsequently provide increasing tumour-to-normal-brain contrast as radioactivity decreases from normal brain whereas it is relatively better retained in tumour tissue. In this regard, [^18^F]FPBT demonstrated two potentially useful characteristics: early brain uptake and a subsequent increase in tumour-to-brain contrast. The rapid early brain entry of [^18^F]FPBT is also consistent with the broader principle that central nervous system exposure is governed by physicochemical and biological processes operating at the blood–brain interface.^46^ The observation that BTPB also showed increasing tumour-to-brain contrast, although over a later observation period, provides a rationale for further investigation of [^18^F]FPBT and related radiolabelled analogues for imaging the intracranial distribution characteristics of BTPB. More broadly, these findings suggest that preservation of early brain access, preferential clearance from normal brain, and relative tumour retention are important distribution characteristics to consider in the further development and optimisation of BTPB-derived imaging probes.

Nevertheless, several limitations preclude a direct pharmacokinetic comparison. [^18^F]FPBT and BTPB were evaluated using substantially different administered masses (tracer *versus* pharmacological doses), analytical methods (γ-counting *versus* ICP-MS), and sampling intervals (10–30 min *versus* 2–4 h). Differences in administered mass may themselves influence tissue distribution and clearance; therefore, even structurally related compounds cannot be assumed to exhibit identical pharmacokinetics under tracer- and pharmacological-dose conditions. Furthermore, the orthotopic biodistribution study of [^18^F]FPBT was performed with a limited number of animals (*n* = 2), and these findings should therefore be regarded as preliminary and descriptive. Accordingly, the present results neither establish pharmacokinetic equivalence nor validate [^18^F]FPBT as a quantitative surrogate for BTPB.

Further studies using matched sampling times, comparable experimental conditions, and adequately powered animal cohorts are required to determine the extent to which [^18^F]FPBT-derived PET measurements reflect the intracranial distribution of BTPB-derived boron. Such studies should directly correlate PET-derived [^18^F]FPBT uptake with tissue boron concentrations and determine whether tracer-derived tumour-to-brain contrast can predict the corresponding distribution of BTPB. Ultimately, quantitative application to BNCT treatment planning would require validation of the relationship between imaging-derived parameters and tissue boron concentrations, followed by appropriate conversion of spatial and temporal distribution data into absorbed-dose estimates according to established dosimetric principles.^47,48^

## 4. Conclusions

In this study, we established radiohalogenation strategies for the BNCT agent BTPB and systematically compared the radiochemical and biological characteristics of its iodine-125- and fluorine-18-labelled analogues. Copper-mediated iododeboronation enabled the efficient synthesis of [^125^I]IPBT, whereas copper-mediated nucleophilic radiofluorination afforded [^18^F]FPBT after systematic optimisation of Cu(OTf)_2_/pyridine loading, reaction time, and temperature. Although high reaction-stage conversion was achieved under the selected radiofluorination conditions, the overall isolated yield of [^18^F]FPBT remained modest, reflecting radioactivity losses during purification and subsequent solvent evaporation and indicating the need for further optimisation of isolation and formulation procedures. Both radiolabelled analogues exhibited high radiochemical purity, adequate molar activity, moderate lipophilicity, and favourable *in vitro* radiolabel stability. Despite these broadly similar physicochemical characteristics, marked differences were observed in their *in vivo* distribution profiles.

Comparative biodistribution and small-animal imaging demonstrated distinct *in vivo* behaviours of the two radiohalogenated analogues. [^18^F]FPBT exhibited early brain uptake and a time-dependent increase in tumour-to-brain contrast, whereas [^125^I]IPBT showed a distinct organ distribution pattern together with findings consistent with possible *in vivo* deiodination. In the orthotopic glioblastoma model, [^18^F]FPBT and BTPB showed a qualitatively similar directional change toward increased tumour-to-brain contrast within their respective observation periods. However, because the two compounds were evaluated using different administered masses, analytical methods, and sampling intervals, this observation should be interpreted as a qualitative similarity in intracranial distribution behaviour rather than evidence of pharmacokinetic correspondence.

Collectively, these findings demonstrate that radiohalogen substitution can substantially influence the *in vivo* behaviour of BTPB-derived analogues despite their broadly similar *in vitro* characteristics. The present results support further investigation of [^18^F]FPBT as a candidate PET imaging analogue of BTPB rather than as a validated pharmacokinetic surrogate. In particular, early brain access, preferential reduction of activity in normal brain relative to tumour tissue, and the resulting increase in tumour-to-brain contrast represent potentially relevant distribution characteristics for the further development of BTPB-derived imaging probes. Further studies using matched sampling times, comparable experimental conditions, adequately powered animal cohorts, and direct correlation between PET-derived uptake and tissue boron concentrations will be required to determine whether [^18^F]FPBT can quantitatively reflect BTPB-derived boron distribution. More broadly, this study highlights the importance of systematic radiohalogen selection and rigorous *in vivo* evaluation in the development of radiolabelled analogues of boron-based BNCT agents.

## Author contributions

C. I.: investigation, data curation, and writing – original draft. M. R. Z.: investigation, data curation, and writing – original draft, and animal model preparation. I. B.: investigation, including animal model preparation and PET and SPECT image acquisition. K. J. K.: investigation, including PET and SPECT image acquisition. J. Y. K.: investigation and formal analysis. H. Y. K. and J.-A. P.: writing – review and editing. All authors have read and agreed to the final version of the manuscript.

## Conflicts of interest

There are no conflicts to declare.

## Supplementary Material

RA-OLF-D6RA07719F-s001

## Data Availability

The data supporting this article have been included in the main manuscript and its supplementary information (SI). Supplementary information: chemical characterisation data, radiolabelling optimisation results, analytical calibration curves, physicochemical property predictions, cellular uptake and subcellular distribution data, and supplementary biodistribution results. See DOI: https://doi.org/10.1039/d6ra07719f.

## References

[cit1] Barth R. F., Zhang Z., Liu T. (2018). A realistic appraisal of boron neutron capture therapy as a cancer treatment modality. Cancer Commun..

[cit2] Moss R. L. (2014). Critical review, with an optimistic outlook, on boron neutron capture therapy (BNCT). Appl. Radiat. Isot..

[cit3] Islam M. M., Lertnaisat P., Meesungnoen J., Sanguanmith S., Jay-Gerin J.-P., Katsumura Y., Mukai S., Umehara R., Shimizu Y., Suzuki M. (2017). Monte Carlo track chemistry simulations of the radiolysis of water induced by the recoil ions of the ^10^B(n, α)^7^Li nuclear reaction. 1. Calculation of the yields of primary species up to 350 °C. RSC Adv..

[cit4] Wang W., Zhang E., Shan J., Zhang M., Cai R., Li R., Pang L., Li B., Zang D. (2026). State-of-the-art boron clusters for boron neutron-capture therapy. Theranostics.

[cit5] Xu H., Liu J., Li R., Lin J., Gui L., Wang Y., Jin Z., Xia W., Liu Y., Cheng S., Yuan Z. (2024). Novel promising boron agents for boron neutron capture therapy: current status and outlook on the future. Coord. Chem. Rev..

[cit6] Coghi P., Li J., Hosmane N. S., Zhu Y. (2023). Next generation of boron neutron capture therapy (BNCT) agents for cancer treatment. Med. Res. Rev..

[cit7] Sauerwein W. A. G., Igawa K., Hérault J., Fossati P., Altieri S., Moss R. L., Wittig A. (2024). Hadron therapy in radiation oncology and why BNCT is a paradigm shift. Health Technol..

[cit8] Fukuda H. (2021). Boron neutron capture therapy (BNCT) for cutaneous malignant melanoma using ^10^B-p-boronophenylalanine (BPA) with special reference to the radiobiological basis and clinical results. Cells.

[cit9] Monti Hughes A., Hu N. (2023). Optimizing boron neutron capture therapy (BNCT) to treat cancer: an updated review on the latest developments on boron compounds and strategies. Cancers.

[cit10] Zheng X., Pan J., Lin D., Shao W. (2025). L-type amino acid transporter 1 in enhancing boron neutron capture therapy: mechanisms, challenges and future directions. Int. J. Mol. Med..

[cit11] Achmad A., Hanaoka H., Holik H. A., Endo K., Tsushima Y., Kartamihardja A. H. S. (2025). LAT1-specific PET radiotracers: development and clinical experiences of a new class of cancer-specific radiopharmaceuticals. Theranostics.

[cit12] Ishiwata K. (2019). 4-Borono-2-[^18^F]fluoro-L-phenylalanine PET for boron neutron capture therapy-oriented diagnosis: overview of a quarter century of research. Ann. Nucl. Med..

[cit13] Kondo N., Hirano F., Kanai Y., Suzuki K., Miyazaki A., Temma T. (2026). A LAT1-selective PET tracer, 5-[^18^F]F-αMe-3BPA, as a companion to its structurally matched ^10^B analog in boron neutron capture therapy. Eur. J. Nucl. Med. Mol. Imaging.

[cit14] Fukumitsu N., Matsumoto Y. (2021). Development of an imaging technique for boron neutron capture therapy. Cells.

[cit15] Jin W. H., Seldon C., Butkus M., Sauerwein W., Giap H. B. (2022). A review of boron neutron capture therapy: its history and current challenges. Int. J. Part. Ther..

[cit16] Yang J.-u., Kim S., Lee K. C., Lee Y. J., Kim J. Y., Park J.-A. (2022). Development of brain-tumor-targeted benzothiazole-based boron complex for boron neutron capture therapy. ACS Med. Chem. Lett..

[cit17] Tredwell M., Preshlock S. M., Taylor N. J., Gruber S., Huiban M., Passchier J., Mercier J., Génicot C., Gouverneur V. (2014). A general copper-mediated nucleophilic ^18^F fluorination of arenes. Angew. Chem., Int. Ed..

[cit18] Mossine A. V., Brooks A. F., Makaravage K. J., Miller J. M., Ichiishi N., Sanford M. S., Scott P. J. H. (2015). Synthesis of [^18^F]arenes *via* the copper-mediated [^18^F]fluorination of boronic acids. Org. Lett..

[cit19] Andrews M. J., Carpentier A., Slawin A. M. Z., Cordes D. B., Macgregor S. A., Watson A. J. B. (2023). Mechanism of Cu-catalyzed iododeboronation: a description of ligand-enabled transmetalation, disproportionation, and turnover in Cu-mediated oxidative coupling reactions. ACS Catal..

[cit20] Kondo Y., Kimura H., Tanaka M., Hattori Y., Kawashima H., Takahashi K., Yasui H. (2024). Mechanistic insights into the effect of sodium iodide on copper-mediated iododeboronation. Chem. – Eur. J..

[cit21] Kondo Y., Kimura H., Sasaki M., Koike S., Yagi Y., Hattori Y., Kawashima H., Yasui H. (2023). Effect of water on direct radioiodination of small molecules/peptides using copper-mediated iododeboronation in water-alcohol solvent. ACS Omega.

[cit22] Rong J., Haider A., Jeppesen T. E., Josephson L., Liang S. H. (2023). Radiochemistry for positron emission tomography. Nat. Commun..

[cit23] Cavina L., van der Born D., Klaren P. H. M., Feiters M. C., Boerman O. C., Rutjes F. P. J. T. (2017). Design of radioiodinated pharmaceuticals: structural features affecting metabolic stability towards *in vivo* deiodination. Eur. J. Org. Chem..

[cit24] Pandurang T. P., Cacaccio J., Durrani F. A., Dukh M., Alsaleh A. Z., Sajjad M., D'Souza F., Kumar D., Pandey R. K. (2023). A remarkable difference in pharmacokinetics of fluorinated *versus* iodinated photosensitizers derived from chlorophyll-a and a direct correlation between the tumor uptake and anti-cancer activity. Molecules.

[cit25] Loaiza-Pérez A. I., Trapani V., Hose C., Singh S. S., Trepel J. B., Stevens M. F. G., Bradshaw T. D., Sausville E. A. (2002). Aryl hydrocarbon receptor mediates sensitivity of MCF-7 breast cancer cells to antitumor agent 2-(4-amino-3-methylphenyl)benzothiazole. Mol. Pharmacol..

[cit26] Zhang J., Cui S., Shen L., Gao Y., Liu W., Zhang C., Zhuang S. (2022). Promotion of bladder cancer cell metastasis by 2-mercaptobenzothiazole *via* its activation of aryl hydrocarbon receptor transcription: molecular dynamics simulations, cell-based assays, and machine learning-driven prediction. Environ. Sci. Technol..

[cit27] Serdons K., Verduyckt T., Vanderghinste D., Cleynhens J., Borghgraef P., Vermaelen P., Terwinghe C., Van Leuven F., Van Laere K., Kung H., Bormans G., Verbruggen A. (2009). Synthesis of ^18^F-labelled 2-(4′-fluorophenyl)-1,3-benzothiazole and evaluation as amyloid imaging agent in comparison with [^11^C]PIB. Bioorg. Med. Chem. Lett..

[cit28] Ertl P., Rohde B., Selzer P. (2000). Fast calculation of molecular polar surface area as a sum of fragment-based contributions and its application to the prediction of drug transport properties. J. Med. Chem..

[cit29] Daina A., Zoete V. (2016). A boiled-egg to predict gastrointestinal absorption and brain penetration of small molecules. ChemMedChem.

[cit30] Daina A., Michielin O., Zoete V. (2017). SwissADME: a free web tool to evaluate pharmacokinetics, drug-likeness and medicinal chemistry friendliness of small molecules. Sci. Rep..

[cit31] Diao X., Shang Q., Guo M., Huang Y., Zhang M., Chen X., Liang Y., Sun X., Zhou F., Zhuang J., Liu S. J., Vogel C. F. A., Rastinejad F., Wu D. (2025). Structural basis for the ligand-dependent activation of heterodimeric AHR-ARNT complex. Nat. Commun..

[cit32] Brauze D., Zawierucha P., Kiwerska K., Bednarek K., Oleszak M., Rydzanicz M., Jarmuz-Szymczak M. (2017). Induction of expression of aryl hydrocarbon receptor-dependent genes in human HepaRG cell modified by shRNA and treated with β-naphthoflavone. Mol. Cell. Biochem..

[cit33] Vegt E., de Jong M., Wetzels J. F. M., Masereeuw R., Melis M., Oyen W. J. G., Gotthardt M., Boerman O. C. (2010). Renal toxicity of radiolabeled peptides and antibody fragments: mechanisms, impact on radionuclide therapy, and strategies for prevention. J. Nucl. Med..

[cit34] Hernández-Lozano I., Mairinger S., Filip T., Löbsch M., Stanek J., Kuntner C., Bauer M., Zeitlinger M., Hacker M., Helbich T. H., Wanek T., Langer O. (2023). Positron emission tomography-based pharmacokinetic analysis to assess renal transporter-mediated drug-drug interactions of antimicrobial drugs. Antimicrob. Agents Chemother..

[cit35] Ogawa K., Takeda T., Yokokawa M., Yu J., Makino A., Kiyono Y., Shiba K., Kinuya S., Odani A. (2018). Comparison of radioiodine- or radiobromine-labeled RGD peptides between direct and indirect labeling methods. Chem. Pharm. Bull..

[cit36] Petrov S. A., Yusubov M. S., Beloglazkina E. K., Nenajdenko V. G. (2022). Synthesis of radioiodinated compounds. Classical approaches and achievements of recent years. Int. J. Mol. Sci..

[cit37] Wu D., Chen Q., Chen X., Han F., Chen Z., Wang Y. (2023). The blood–brain barrier: structure, regulation and drug delivery. Signal Transduction Targeted Ther..

[cit38] Terstappen G. C., Meyer A. H., Bell R. D., Zhang W. (2021). Strategies for delivering therapeutics across the blood–brain barrier. Nat. Rev. Drug Discovery.

[cit39] Puris E., Gynther M., Auriola S., Huttunen K. M. (2020). L-type amino acid transporter 1 as a target for drug delivery. Pharm. Res..

[cit40] Costa I. M., Cheng J., Osytek K. M., Imberti C., Terry S. Y. A. (2021). Methods and techniques for *in vitro* subcellular localization of radiopharmaceuticals and radionuclides. Nucl. Med. Biol..

[cit41] Zheng N., Tsai H. N., Zhang X., Rosania G. R. (2011). The subcellular distribution of small molecules: from pharmacokinetics to synthetic biology. Mol. Pharm..

[cit42] Li J., Shi Y., Zhang Z., Liu H., Lang L., Liu T., Chen X., Liu Z. (2019). A metabolically stable boron-derived tyrosine serves as a theranostic agent for positron emission tomography guided boron neutron capture therapy. Bioconjugate Chem..

[cit43] Bibi I., Kang K. J., Kim J. Y., Mushtaq S., Park J.-A. (2025). Development of structurally identical therapeutic and diagnostic agents for image-guided boron neutron capture therapy: c(RGD-BPA-K) peptide with ^125^I/^nat^I albumin-binding moiety. Mol. Pharm..

[cit44] Hu N., Tanaka H., Kakino R., Yoshikawa S., Miyao M., Akita K., Isohashi K., Aihara T., Nihei K., Ono K. (2021). Evaluation of a treatment planning system developed for clinical boron neutron capture therapy and validation against an independent Monte Carlo dose calculation system. Radiat. Oncol..

[cit45] Skwierawska D., López-Valverde J. A., Balcerzyk M., Leal A. (2022). Clinical viability of boron neutron capture therapy for personalized radiation treatment. Cancers.

[cit46] Banks W. A. (2016). From blood–brain barrier to blood–brain interface: new opportunities for CNS drug delivery. Nat. Rev. Drug Discovery.

[cit47] Bolch W. E., Eckerman K. F., Sgouros G., Thomas S. R. (2009). MIRD pamphlet no. 21: a generalized schema for radiopharmaceutical dosimetry—standardization of nomenclature. J. Nucl. Med..

[cit48] O'Donoghue J., Zanzonico P., Humm J., Kesner A. (2022). Dosimetry in radiopharmaceutical therapy. J. Nucl. Med..

