# Supplementary material for: Development and comparative pharmacokinetic evaluation of iodine-125- and fluorine-18-labelled analogues of 4-(benzo[d]thiazol-2-yl)phenylboronic acid for boron neutron capture therapy
Source: RSC Adv. 2026 Nov 2;16(49):67600–12. doi: 10.1039/d6ra07719f (PMC13725790; doi:10.1039/d6ra07719f)
Supplement: Supplementary file 1 [file RA-016-D6RA07719F-s001.pdf]

Supplementary Information (SI) for RSC Advances.
This journal is © The Royal Society of Chemistry

Supplementary Information

Development and comparative pharmacokinetic evaluation of iodine-125- and fluorine-18-labelled analogues of 4-(benzo[d]thiazol-2-yl)phenylboronic acid for boron neutron capture therapy

Changkeun Im[1,†], Mah Rukh Zia[1,2,†], Iqra Bibi[1,2], Kyung Jun Kang[1], Jung Young Kim[1,2], Ho Young Kim[1,2,*], Ji-Ae Park[1,2,*]

[1] *Division of Applied RI, Korea Institute of Radiological & Medical Sciences (KIRAMS), 75 Nowon-ro, Nowon-gu, Seoul 01812, Republic of Korea*

[2] *Radiological and Medico-Oncological Sciences, University of Science and Technology (UST), 217, Gajeong-ro, Yuseong-gu, Daejeon 34113, Republic of Korea*

*Corresponding authors: jpark@kirams.re.kr and areas2001@kirams.re.kr

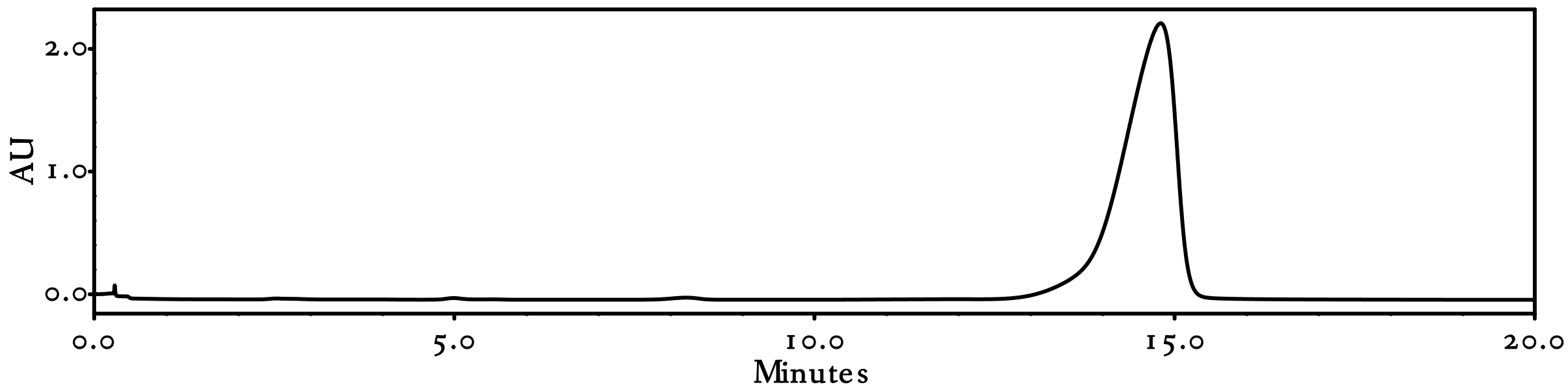

Figure S1. Analytical reversed-phase HPLC chromatogram of purified 2-(4-iodophenyl)benzo[d]thiazole (IPBT , Method A). The compound eluted at a retention time (R_t) of 14.79 min, confirming chemical purity prior to radiochemical comparison studies.

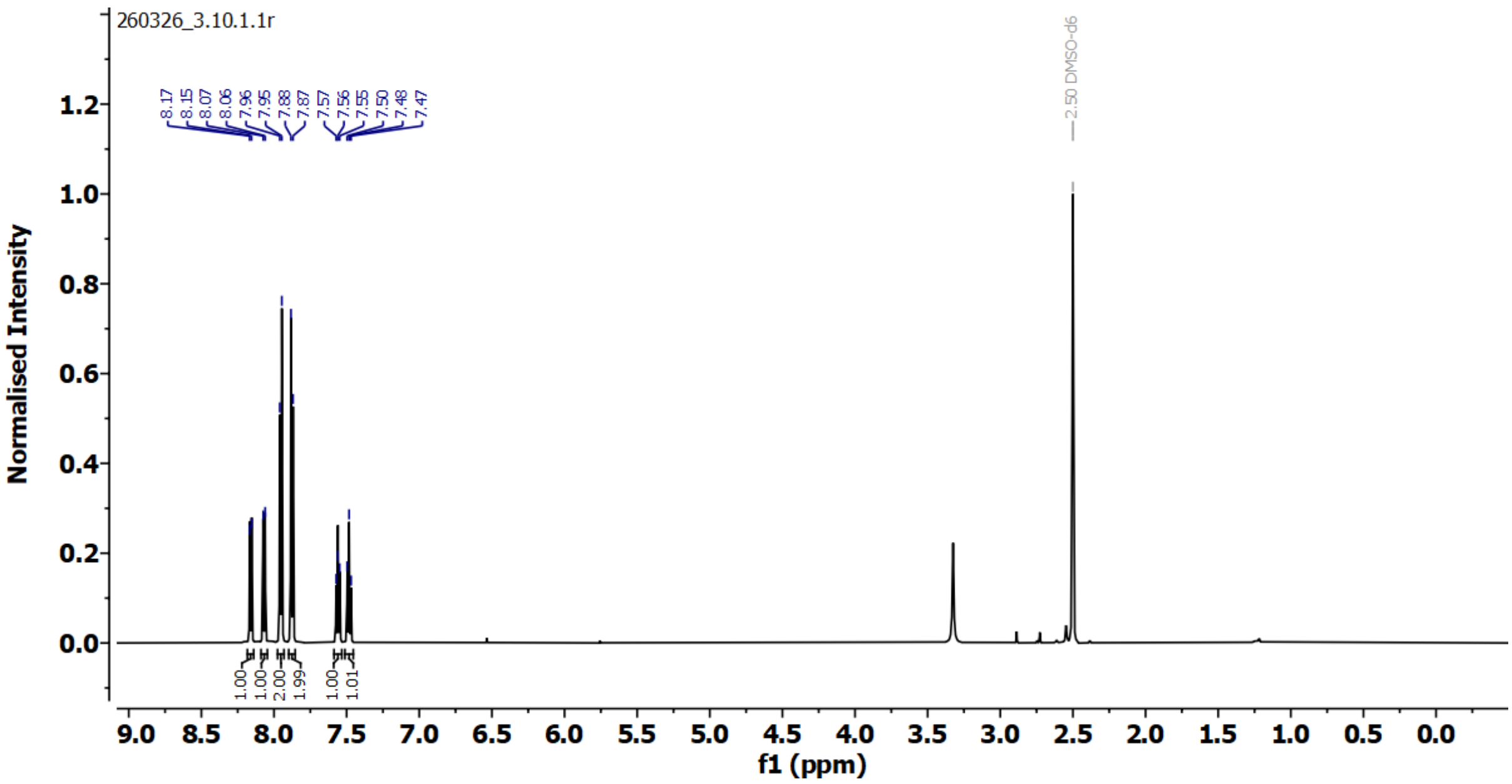

Figure S2. ^{1}H NMR spectrum of 2-(4-iodophenyl)benzo[d]thiazole (IPBT) recorded in DMSO-d_6 at 600 MHz.

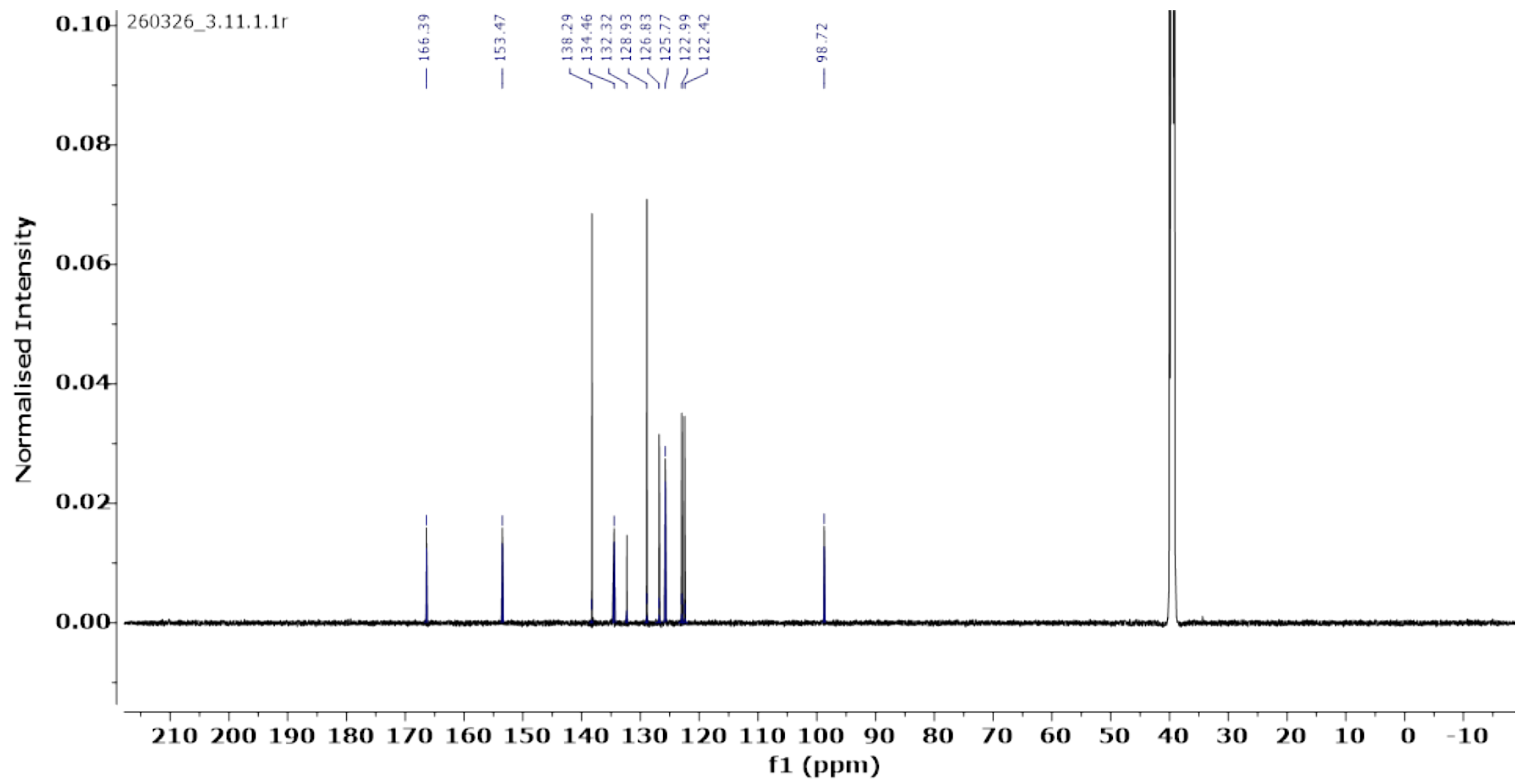

Figure S3. ^{13}C NMR spectrum of 2-(4-iodophenyl)benzo[d]thiazole (IPBT) recorded in DMSO-d_6 at 150 MHz

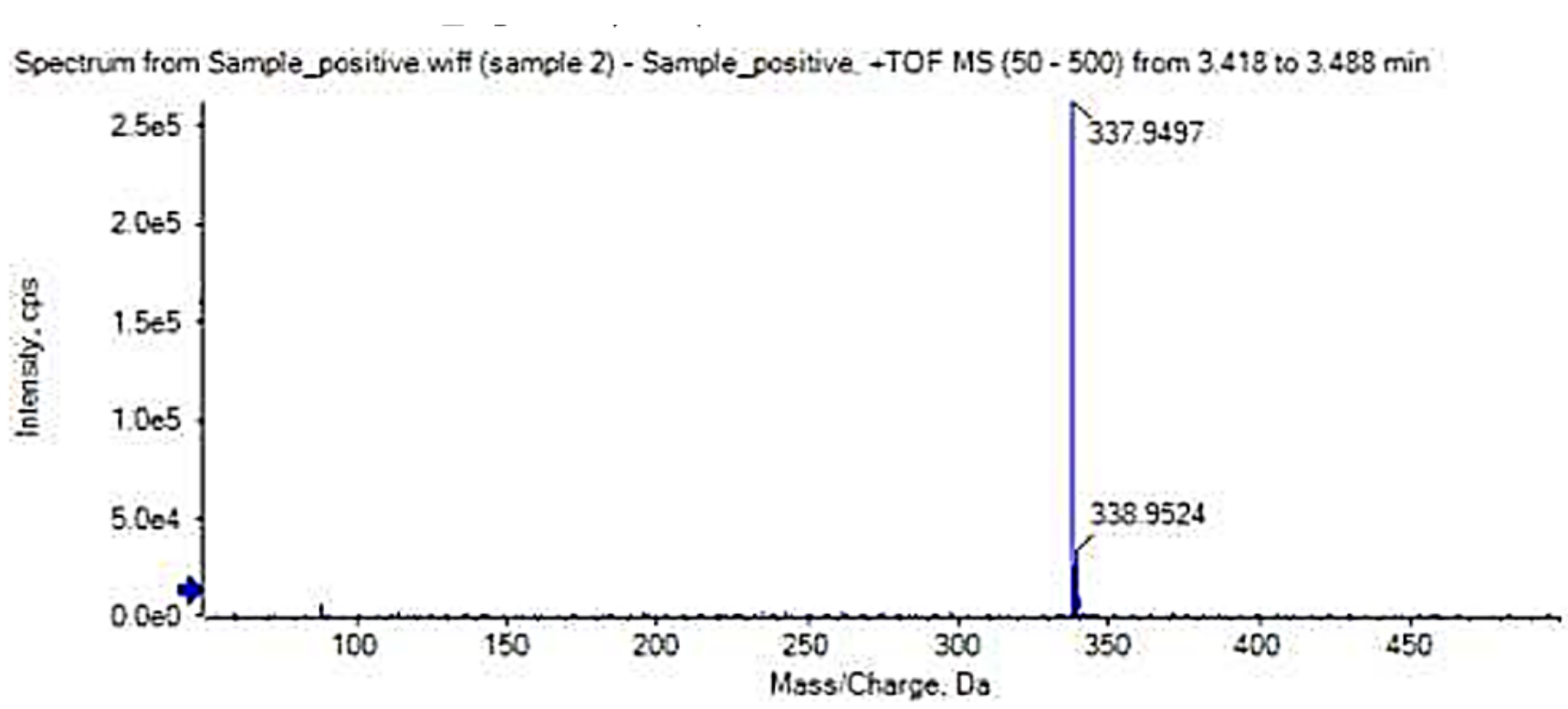

Figure S4. High-resolution electrospray ionization mass spectrum (HRMS, ESI⁺) of 2-(4-iodophenyl)benzo[d]thiazole (IPBT).

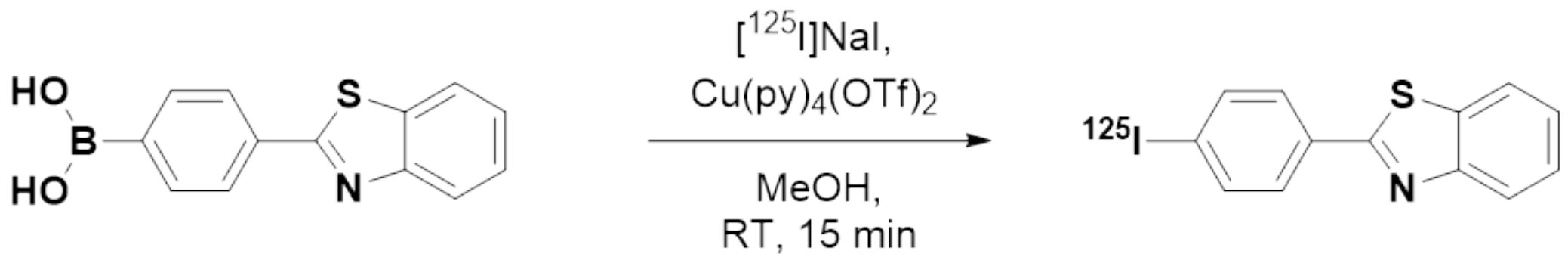

Entry	Substrate (μmol)	Reagents (μmol)	Solvent[a]	RCC(%)[b]
		$Cu(py)_4(OTf)_2$		
1	0.1	0.02	MeOH	2.5
2	0.1	0.04	MeOH	8.8
3	0.1	0.08	MeOH	69.9
4	0.1	0.11	MeOH	75.9
5	0.1	0.15	MeOH	78.3
6	0.1	0.19	MeOH	77.2

a) Solvent volume was 65 μL; b) RCC(%) was measured based on the silica-TLC (80% Hexane in ethyl acetate); $Cu(py)_4(OTf)_2$: Tetrakis(pyridine)copper(II) triflate

Figure S5. Optimization of copper-mediated radioiodination of BTPB based on radio-TLC analysis. Representative radio-TLC profiles obtained under varying reaction conditions, including copper catalyst loading, solvent, and reaction time. Radiochemical conversion (RCC) was determined by integration of the product and unreacted [^{125}I]iodide fractions. The optimized condition ($Cu(py)_4(OTf)_2$ in MeOH at room temperature for 15 min) afforded the highest RCC toward [^{125}I]IPBT.

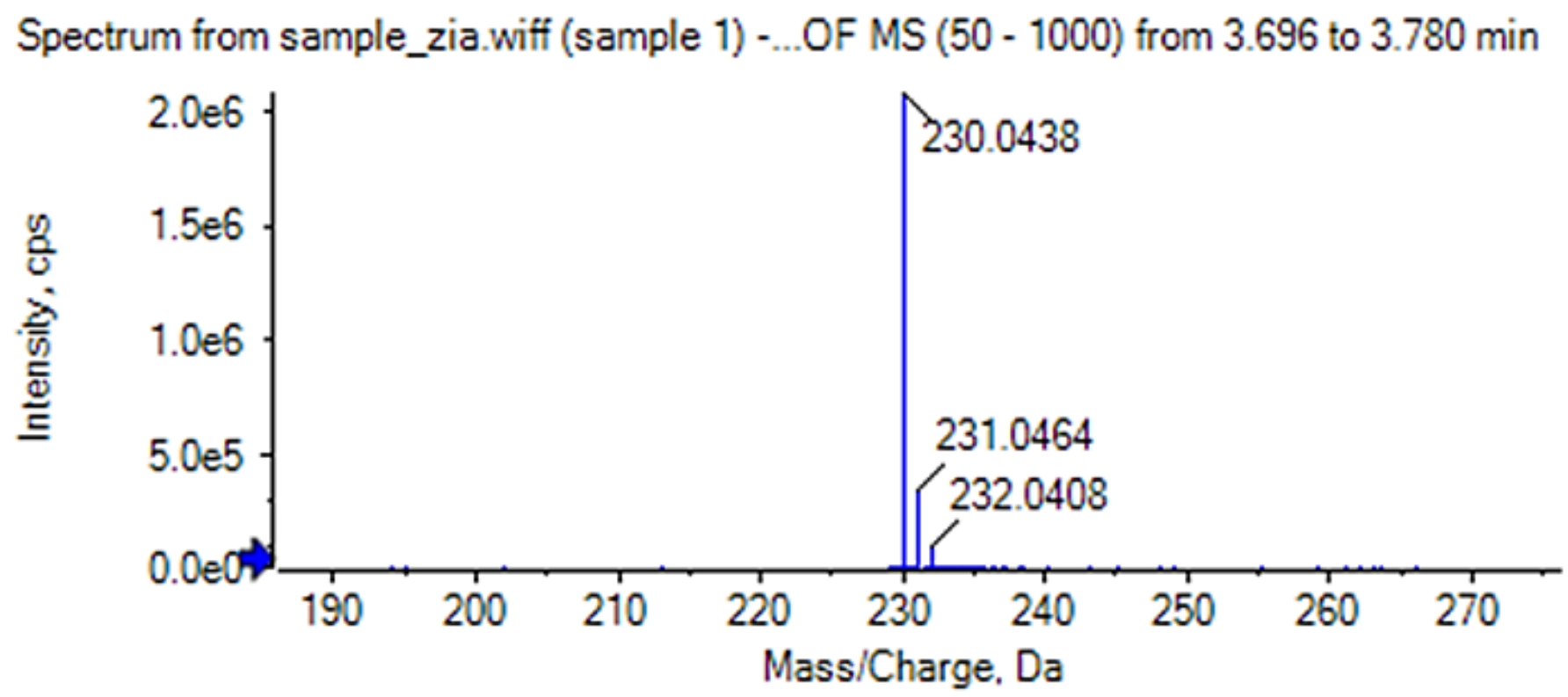

Figure S6. HRMS of 2-(4-fluorophenyl)benzo[d]thiazole (FPBT).

A

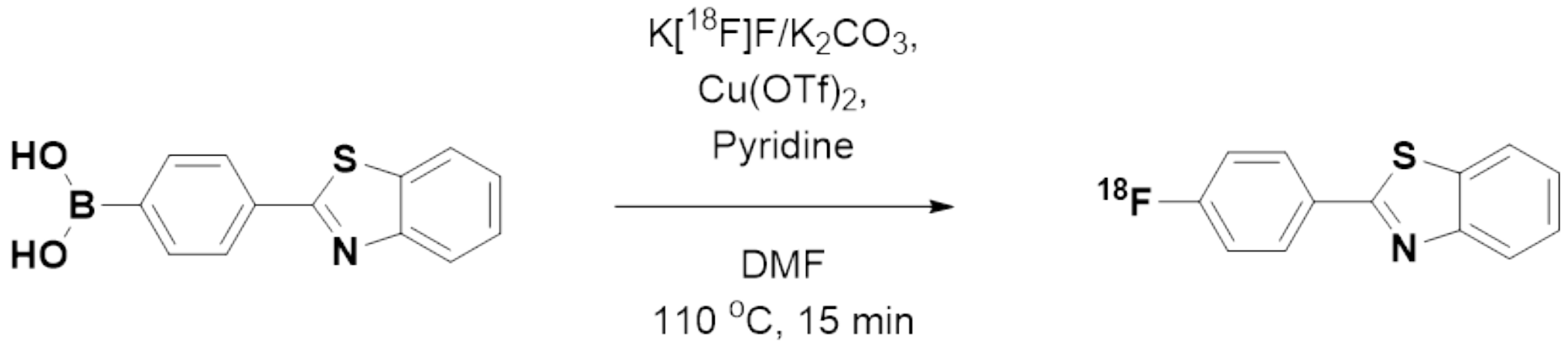

Entry	Substrate (μmol)	Reagents (μmol)		Solvent[a]	RCC(%)[b]
		$Cu(OTf)_2$	Pyridine		
1	4	20	100	DMF	36.0
2	4	20	300	DMF	42.7
3	4	20	400	DMF	30.0
4	4	20	450	DMF	31.0
5	4	20	500	DMF	19.9
6	4	5	300	DMF	67.2
7	4	10	300	DMF	55.3
8	4	30	300	DMF	42.0
9	4	40	300	DMF	37.0
10	4	5	75	DMF	28.4
11	4	5	150	DMF	48.2
12	4	5	300	DMF	67.0
13	4	5	400	DMF	57.0
14	4	5	500	DMF	61.2

a) Solvent volume was 750 μL; b) RCC(%) was measured based on the silica-TLC (80% Hexane in ethyl acetate); $Cu(OTf)_2$: Copper(II) trifluoromethanesulfonate

B

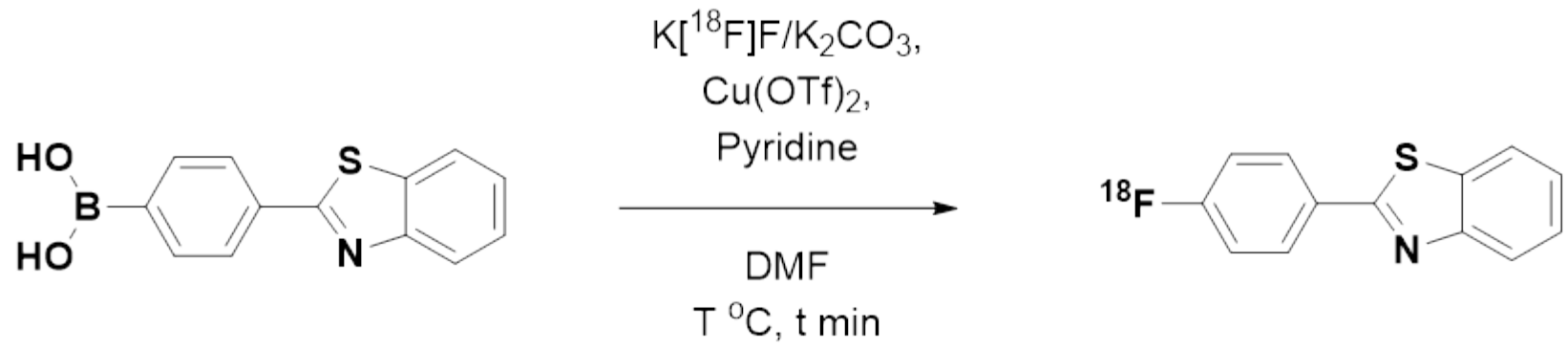

Entry	Substrate (μmol)	Reagents (μmol)		Solvent[a]	Time (min)	Temperature (°C)	RCC(%)[b]
		$Cu(OTf)_2$	Pyridine				
1	4	5	300	DMF	5	110	74.17
2	4	5	300	DMF	10	110	77.61
3	4	5	300	DMF	15	110	80.07
4	4	5	300	DMF	20	110	80.21
5	4	5	300	DMF	25	110	69.24
6	4	5	300	DMF	30	110	59.60
7	4	5	300	DMF	15	70	46.53
8	4	5	300	DMF	15	90	58.35
9	4	5	300	DMF	15	110	76.76
10	4	5	300	DMF	15	120	75.05
11	4	5	300	DMF	15	130	75.07
12	4	5	300	DMF	15	150	68.19

a) Solvent volume was 900 μL; b) RCC(%) was measured based on the silica-TLC (80% Hexane in ethyl acetate)

Figure S7. Optimisation of copper-mediated radiofluorination of BTPB for [^{18}F]FPBT radiosynthesis. (A) Effects of $Cu(OTf)_2$ and pyridine loading on radiochemical conversion (RCC) at 110 ℃ for 15 min. (B) Effects of reaction time and temperature on RCC using BTPB (4 μmol), $Cu(OTf)_2$ (5 μmol), and pyridine (300 μmol) in DMF. Reaction time was varied at 110 ℃, whereas reaction temperature was independently varied a fixed reaction time of 15 min. RCC was determined by radio-TLC using silica gel plates with hexane/ethyl acetate (80:20, v/v) as the mobile phase.

Table S1. Stepwise radioactivity recovery [^{18}F]FPBT radiosynthesis, purification, and formulation.

Entry	Pre-HPLC recovery (%)[a]	HPLC product-fraction recovery (%)[b]	C18 SPE recovery (%)[c]	Post-evaporation recovery (%)[d]	Overall isolated RCY (%)[e]
1	39.96	17.14	89.62	27.74	1.50
2	57.99	14.74	88.97	44.14	2.23
3	68.60	11.06	95.83	74.29	3.44
4	49.25	10.12	94.11	67.77	2.38
5	78.65	15.62	94.38	67.49	5.27
6	50.71	16.25	92.00	60.09	3.80
Mean ± SD	57.53 ± 14.09	14.15 ± 2.89	92.49 ± 2.77	56.92 ± 17.64	3.10 ± 1.35

a) Pre-HPLC recovery was calculated from the decay-corrected activity injected onto the preparative HPLC system relative to the starting activity; b) HPLC product-fraction recovery was calculated from the decay-corrected activity recovered in the [^{18}F]FPBT-containing fraction relative to the activity injected onto the preparative HPLC system and reflects product-fraction recovery rather than chromatographic recovery alone; c) C18 SPE recovery was calculated from the decay-corrected activity recovered after C18 elution relative to the activity loaded onto the cartridge; d) Post-evaporation recovery was calculated as the decay-corrected activity remaining after ethanol evaporation relative to the activity recovered in the C18 SPE eluate; e) Overall isolated RCY was calculated from the decay-corrected activity of the final formulated product relative to the starting activity. All data are presented as mean ± sample standard deviation (SD, n = 6)

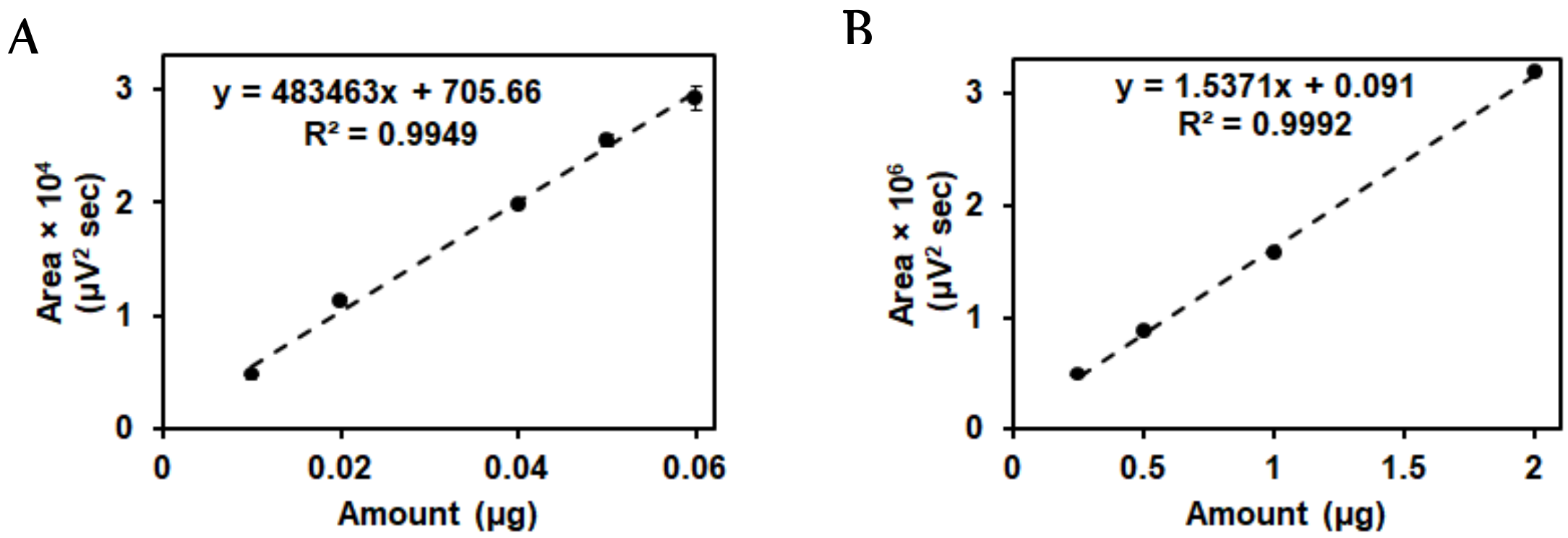

Figure S8. Analytical Calibration curve of (A) IPBT and (B) FPBT. Peak areas obtained from UV detection were plotted against the corresponding injected amounts. Linear regression analysis demonstrated good linearity over the tested concentration range. Data are presented as mean ± SD (n = 3).

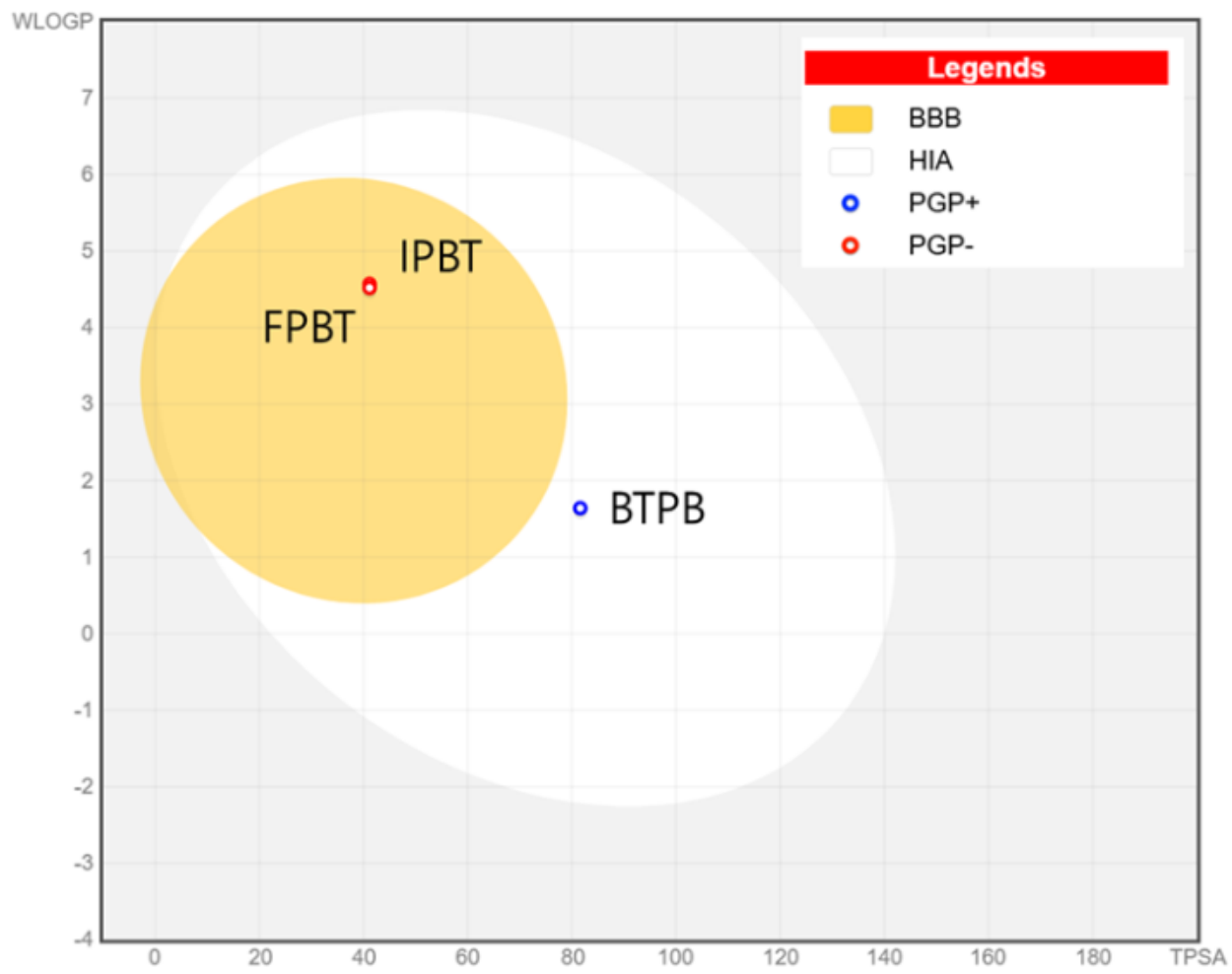

Figure S9. Plot of WLOGP versus TPSA showing predicted blood-brain barrier (BBB) permeability and human intestinal absorption (HIA) regions. The yellow area represents the BBB-permeable space, and the white are indicates the HIA space. FPBT, IPBT, and BTPB are displayed at their respective positions; blue and red circles denote P-glycoprotein (PGP) substrates and non-substrates, respectively.

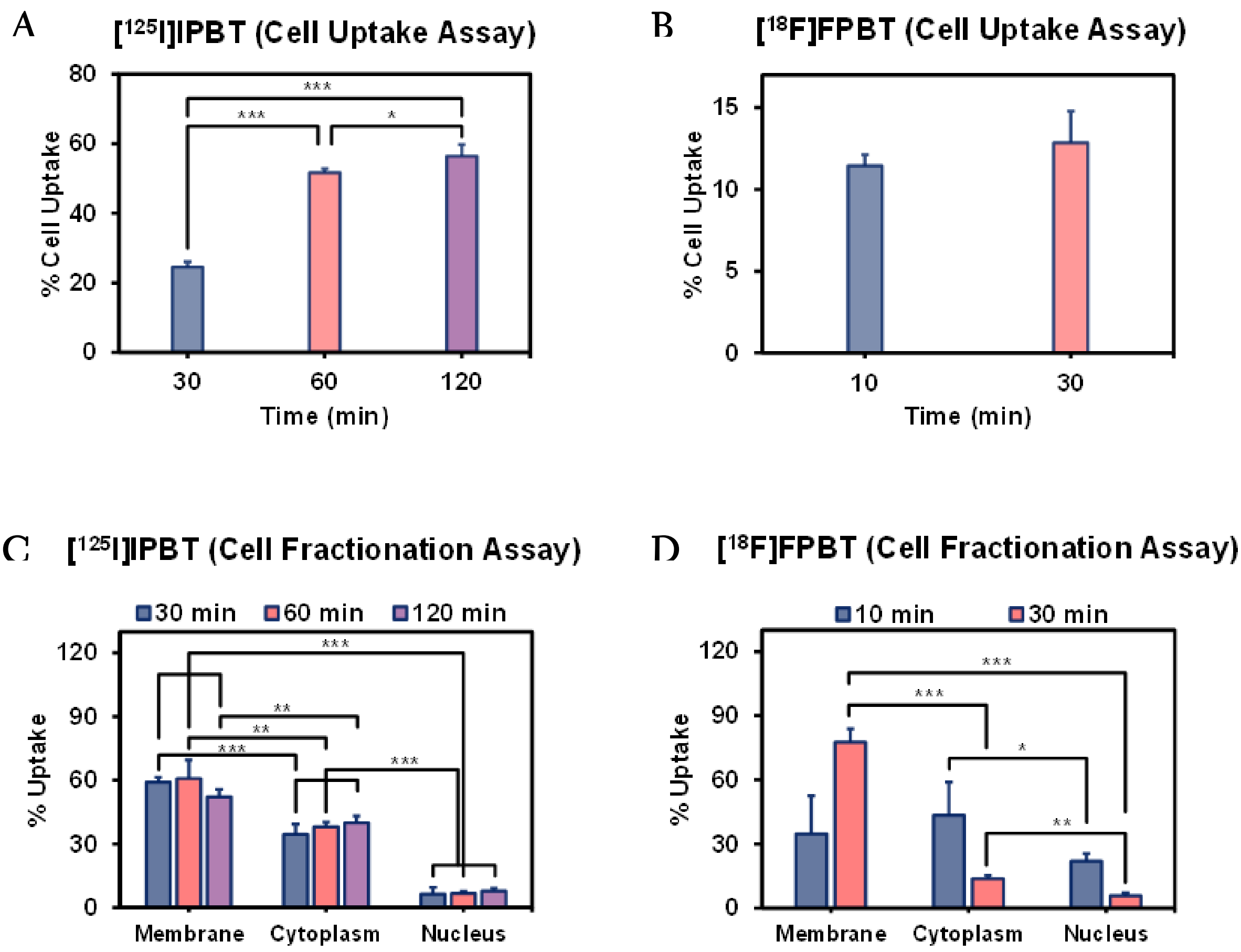

Figure S10. Cellular association and intracellular distribution of BTPB and its radiolabeled probes. Time-dependent cellular association of (A) [^{125}I]IPBT after 30, 60, and 120 min of incubation, and (B) [^{18}F]FPBT after 10 and 30 min of incubation. Intracellular distribution of (C) [^{125}I]IPBT at after 30, 60, and 120 min of incubation, and (D) [^{18}F]FPBT after 10 and 30 min of incubation. Data are presented as mean ± SD.

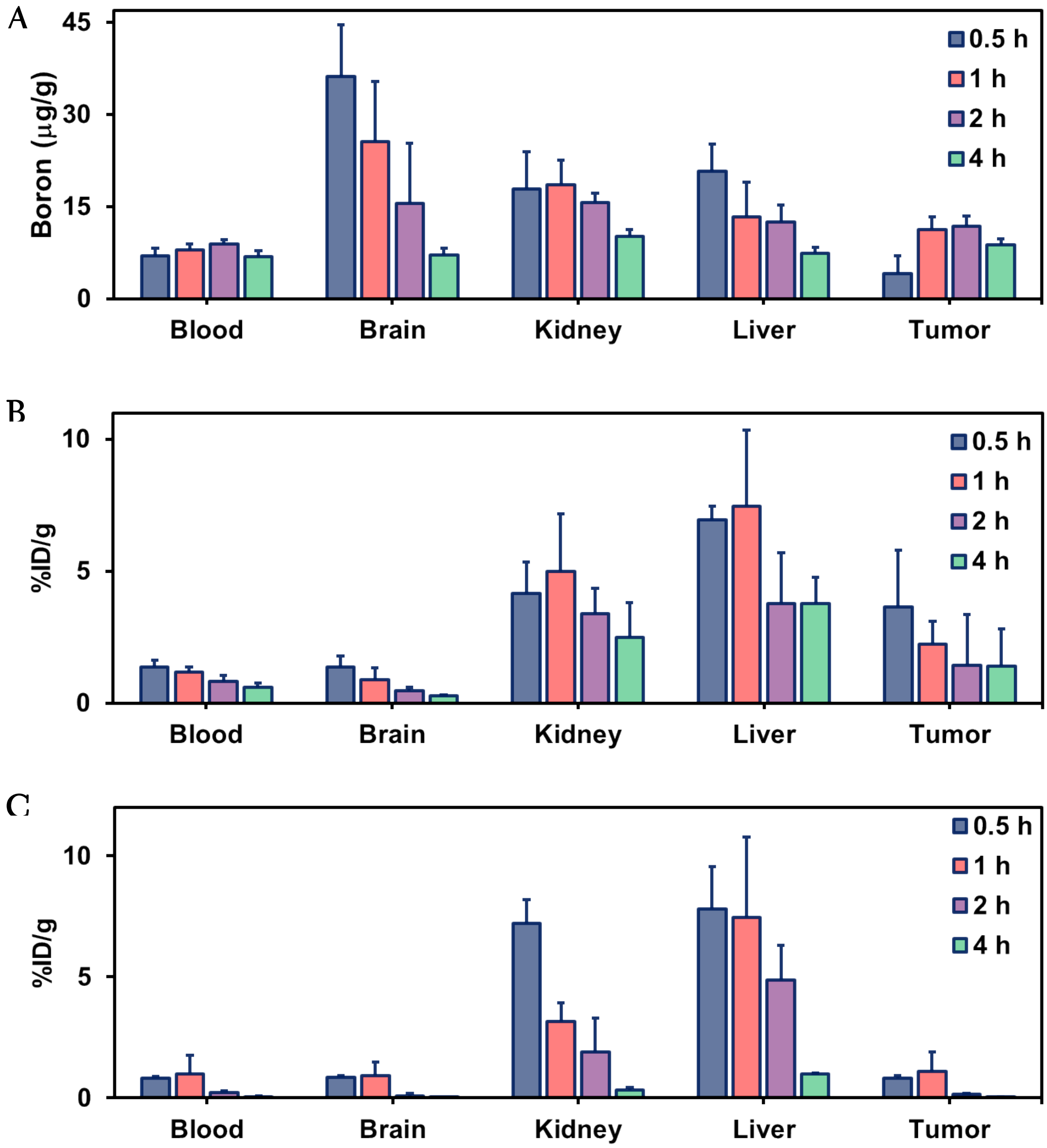

Figure S11. Biodistribution data of (A) BTPB, (B) [^{125}I]IPBT, (C) [^{18}F]FPBT. Measurements were obtained at 0.5, 1, 2, and 4 h p.i. Data are presented as mean ± SD.
